# Public knowledge, safety perceptions, and use patterns of ashwagandha (*Withania somnifera*) among Saudi adults: a mixed-methods study

**DOI:** 10.3389/fphar.2026.1848763

**Published:** 2026-09-18

**Authors:** Amal I. Khalil, Aseel F. Aljohani

**Affiliations:** 1 King Abdullah International Medical Research Center, Jeddah, Saudi Arabia; 2 College of Nursing, King Saud Bin Abdulaziz University for Health Sciences, Jeddah, Saudi Arabia; 3 Ministry of National Guard Health Affairs (MNGHA), Jeddah, Saudi Arabia; 4 Faculty of Nursing, Menoufia University, Shebin ElkomCity, Egypt

**Keywords:** ashwagandha, herbal supplements, mixed methods, pharmacological awareness, safety perception, Saudi Arabia, Withania somnifera

## Abstract

**Background:**

*Withania somnifera*, commonly known as ashwagandha, is a widely used herbal remedy with reported anxiolytic and adaptogenic properties. Despite its growing use globally and regionally, comprehensive public understanding of its pharmacological benefits, safety, and potential risks remains limited within the Saudi Arabian context. This study aimed to evaluate knowledge, safety perceptions, pharmacological awareness, and patterns of ashwagandha use among Saudi adults using a mixed-methods approach.

**Methods:**

A convergent mixed-methods design was employed, incorporating a cross-sectional survey of 508 participants and semi-structured interviews with 20 participants. Data were collected over a 6-month period from August 2025 to January 2026. Quantitative data were analyzed using descriptive statistics, inferential tests, and multiple linear regression analyses to identify predictors of knowledge and attitudes. Qualitative data were thematically analyzed to explore participants’ experiences, motivations, and safety concerns. Quantitative and qualitative findings were integrated during the interpretation phase.

**Results:**

Overall knowledge and pharmacological awareness of ashwagandha were limited, with nearly half of the participants demonstrating low knowledge levels. Social media and informal networks were the primary sources of information, whereas healthcare professionals were rarely consulted. Positive perceptions of ashwagandha were associated with previous use, frequent use, and a self-reported history of anxiety-related symptoms or conditions. Qualitative findings indicated that ashwagandha use was generally sporadic and motivated by stress management. Participants commonly described cautious, self-regulated use because of concerns about side effects and long-term safety. Some participants also reported using ashwagandha concurrently with other herbal supplements or dietary supplements, raising concerns about potential supplement–supplement interactions and cumulative adverse effects.

**Conclusion:**

Ashwagandha use among Saudi adults appears to be influenced primarily by personal experience and psychological factors rather than formal knowledge or professional guidance. Reliance on informal information sources, limited pharmacological awareness, and concerns regarding the safety of combining herbal supplements highlight the need for targeted public health education and greater involvement of healthcare professionals. These findings emphasize the importance of improving public understanding of the pharmacological properties, potential risks, interactions, and safe use of herbal medicines.

## 1 Introduction

Herbal and dietary supplements are increasingly used worldwide, reflecting a growing preference for natural and complementary approaches to health management (World Health Organization, 2019; Ekor, 2014). Among these products, ashwagandha (*Withania somnifera*) has attracted considerable attention because of its proposed adaptogenic, anxiolytic, and anti-inflammatory properties (Lopresti et al., 2019; Langade et al., 2019). Traditionally used in Ayurvedic medicine, ashwagandha is now widely available in international markets, including Saudi Arabia, in various forms such as capsules, powders, extracts, and herbal teas (Salve et al., 2019; Akhgarjand et al., 2022).

From a pharmacological perspective, ashwagandha contains several biologically active compounds, particularly withanolides, which are believed to influence multiple physiological pathways, including the hypothalamic–pituitary–adrenal axis and gamma-aminobutyric acid signaling pathways (Guo and Rezaei, 2024; Mikulska et al., 2023). These mechanisms may contribute to its reported stress-reducing, anxiolytic, and mood-stabilizing effects. Clinical studies and meta-analyses have suggested that ashwagandha supplementation may reduce cortisol levels, improve sleep quality, and alleviate symptoms of anxiety and stress (Wankhede et al., 2015; Björns et al., 2020; Woo et al., 2021). Emerging evidence has also indicated potential benefits for cognitive functioning, metabolic regulation, and physical performance (Almalki et al., 2025; Almech et al., 2024).

Despite these promising findings, important concerns remain regarding the safety, quality, and regulation of ashwagandha products. Reported adverse effects include gastrointestinal disturbances, thyroid dysfunction, and rare cases of liver injury, emphasizing the need for cautious and informed use (Møller et al., 2024; Zaidi et al., 2022; Alasm et al., 2025). The supplement may also interact with commonly prescribed medications, including sedatives, immunosuppressants, antidiabetic medications, antihypertensive medications, and thyroid hormones, potentially resulting in clinically relevant adverse effects or altered therapeutic responses (Saudi Food and Drug Authority, 2022; Başaran et al., 2022).

The regulation and quality control of herbal supplements vary considerably across countries. In many settings, including Saudi Arabia, herbal products may not undergo the same extensive evaluation of efficacy, safety, standardization, and post-marketing surveillance required for conventional pharmaceutical medications (Creswell and Plano Clark, 2018). Variations in product composition, withanolide concentration, ingredient labeling, contamination, and manufacturing quality may further influence their effectiveness and safety. Therefore, consumers may unknowingly use products with inconsistent dosages or insufficient evidence regarding their purity and pharmacological effects.

Globally, herbal supplements are frequently perceived as inherently safe because of their natural origin, despite limited public awareness of their potential adverse effects, contraindications, and drug–supplement interactions (Bergman, 2008; von Elm et al., 2007). In Saudi Arabia, the use of herbal and traditional remedies is deeply rooted in cultural practices and may be influenced by family traditions, social networks, personal experiences, and increasing exposure to digital media (Tong et al., 2007; Patton, 2015). Previous studies conducted in the region have demonstrated the widespread use of herbal medicines; however, public knowledge regarding appropriate dosages, evidence-based benefits, safety precautions, and potential interactions remains inadequate (Etikan et al., 2016; Creswell and Poth, 2018).

Bibliometric analysis can identify influential publications, research trends, collaborative networks, and emerging themes within a scientific field, thereby helping to clarify the development of research and guide future investigations (Özduran et al., 2025). When combined with altmetric indicators, it may also demonstrate how scientific evidence about ashwagandha is disseminated and discussed through social media, news outlets, and other digital platforms (Erkin et al., 2026).

Digital technologies, including social media, YouTube, and artificial intelligence platforms, have become important sources of information about herbal supplements. However, variations in the accuracy, reliability, and scientific quality of online content may contribute to misconceptions regarding the benefits and safety of ashwagandha, highlighting the need to assess public knowledge and safety awareness (Hancı et al., 2023; Palinkas et al., 2015). Existing research on ashwagandha has primarily examined its pharmacological properties and clinical effectiveness, with comparatively limited attention given to public knowledge, safety perceptions, and real-world usage patterns, particularly within specific cultural contexts (Wu et al., 2023). Moreover, studies addressing herbal medicine frequently adopt a broad approach without examining individual herbs and rarely use mixed-methods designs capable of capturing both population-level patterns and personal experiences (Rosenstock et al., 1988). Consequently, there remains an important gap in understanding how individuals perceive the potential benefits and risks of ashwagandha, how they incorporate it into their daily lives, and which social, cultural, psychological, and informational factors influence their decisions to use it.

To address these gaps, the present study employed a convergent mixed-methods design to investigate knowledge, safety perceptions, and usage patterns related to ashwagandha among Saudi adults. By integrating quantitative findings with qualitative experiences, the study sought to provide a comprehensive and culturally grounded understanding of public engagement with this increasingly popular herbal supplement. The findings may inform targeted public health education, support healthcare professionals in providing evidence-based counseling, and promote the safe and responsible use of herbal supplements.

## 2 Significance of the study

The use of ashwagandha (*W. somnifera*) appears to be increasing among people in Saudi Arabia, influenced by the widespread availability of herbal products, cultural acceptance of traditional remedies, social media promotion, and informal recommendations from family members and friends. However, evidence remains limited regarding Saudi adults’ knowledge of its pharmacological effects, appropriate use, potential adverse effects, contraindications, and interactions with medications or other supplements.

This study provides a focused assessment of knowledge, safety perceptions, and patterns of ashwagandha use among Saudi adults by integrating quantitative and qualitative data. It examines how Saudi consumers obtain information about ashwagandha, why they choose to use it, how they perceive its benefits and risks, and whether healthcare professionals are consulted before or during its use.

The findings may identify important knowledge and safety gaps within the Saudi population and clarify the influence of stress, cultural beliefs, previous use of herbal remedies, family and social networks, and digital information sources on consumption decisions. These findings may support the development of culturally appropriate public-health education, strengthen the role of pharmacists, physicians, and nurses in counseling consumers, and promote safer and more evidence-based use of ashwagandha in Saudi Arabia.

## 3 Theoretical framework

Figure 1 presents a comprehensive framework designed utilizing the Health Belief Model in explaining the different elements that shape ashwagandha practice behavior in Saudi Arabia. This framework identifies the relationship between a number of background variables, intermediary health belief factors, and behavioral outcomes in a mixed methods framework. Among the independent variables, sociodemographic factors (age, gender, education, occupation, and region), in addition to the awareness of ashwagandha, information sources, previous experience with herbal supplements, and prior experience with stress or anxiety, are included. Each of these variables can help shape how people think about the advantages and risks associated with using ashwagandha.

**FIGURE 1:**
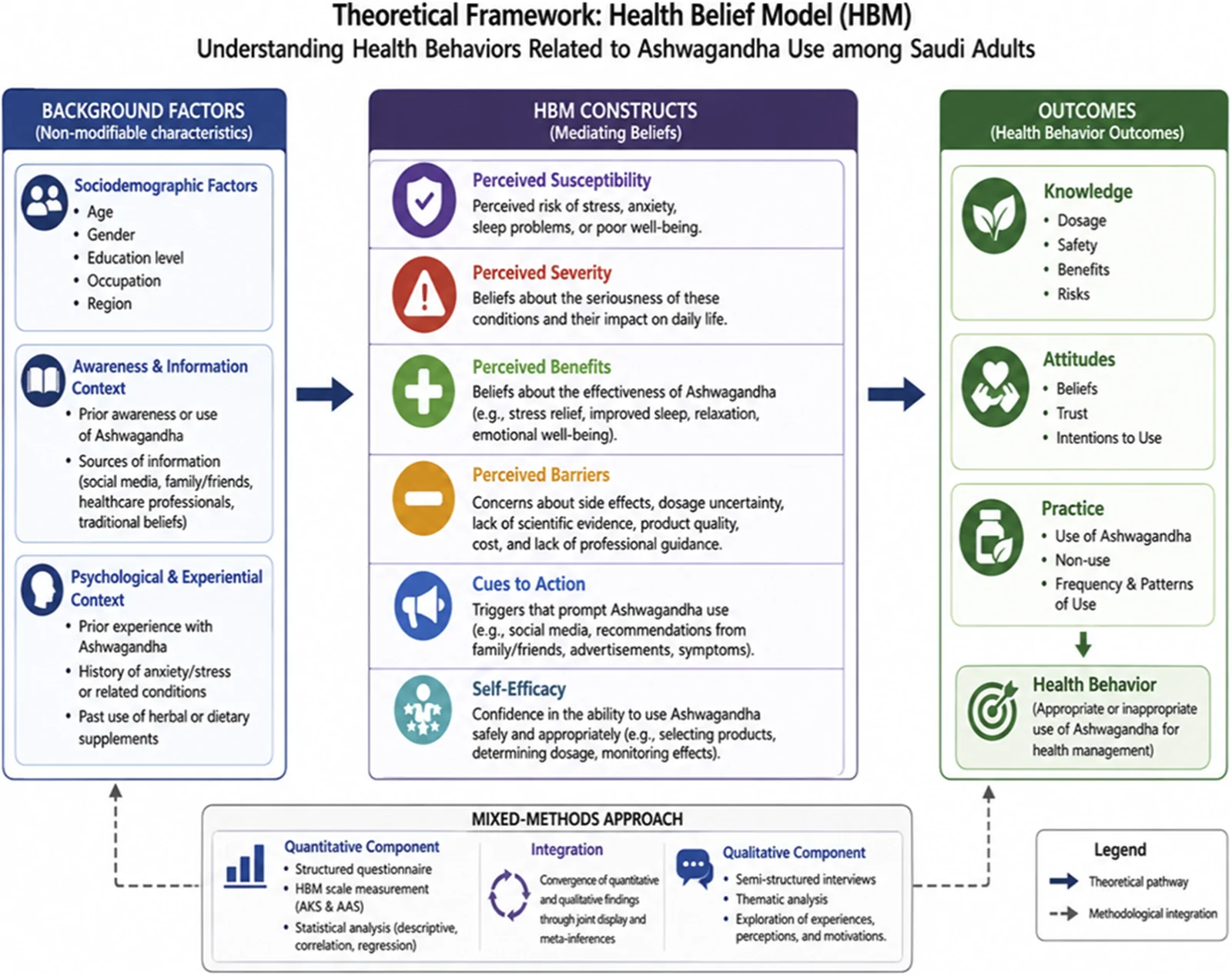
Theoretical framework of the study based on the Health Belief Model (HBM), illustrating the relationship between background factors, mediating beliefs, and health behavior outcomes related to Ashwagandha use among Saudi adults.

The intermediary variables consist of the main constructs found in the Health Belief Model. Perceived susceptibility refers to personal beliefs about one’s susceptibility toward experiencing stress, anxiety, sleep problems, side effects, or interactions when using ashwagandha. Those who believe they are susceptible to stress-related issues are likely to use the herb, while people who think they are susceptible to side effects are likely to abstain from using the herb. Beliefs about the severity of stress, anxiety, insomnia, side effects, or conflicts between medication and supplements relate to perceived severity. The more serious a person thinks stress is, the greater the chances that he/she will consult specialists, initiate treatment, or refrain from using the medications without the supervision of medical professionals. Perceived benefits refer to beliefs that ashwagandha is helpful in managing stress, improving sleep, increasing strength, or facilitating psychological wellbeing. The stronger the belief in these benefits, the stronger the willingness to use the supplement. In contrast, concerns about negative effects, uncertainty about the dosage, lack of knowledge, price, quality of the products, and interaction with drugs or other supplements belong to perceived barriers. These barriers may prevent people from using ashwagandha or make them careful to use it. Cues to action are internal or external stimuli that can trigger ashwagandha-related action. They may include feeling stressed or having difficulties with sleep; receiving recommendations from family and friends; seeing a social media post about ashwagandha and/or its use; seeing advertisements; having previous positive experience or getting medical advice on ashwagandha. This means that the use of ashwagandha can be initiated based on the cues even without sufficient pharmacological knowledge. Self-efficacy is defined as personal confidence in product reliability, dosage selection, adverse effects, and when to consult a physician. Having high levels of self-efficacy may assist in safe advanced usage while no knowledge can lead to inappropriate self-treatment.

Dependent variables under consideration are knowledge, attitude, practice, and health behavior regarding ashwagandha use. Knowledge refers to being aware of indications, dosage, benefits, side effects, contraindications, and interactions. Attitude refers to confidence in the product and its efficiency, perceptions of safety, and readiness to use ashwagandha. Behavior refers to how often and how long a product is used, motives for using it, where it has been obtained from, other supplements which are taken along with ashwagandha and doctor’s recommendations.

The quantitative aspect gives assumptions regarding correlation of background factors, Health Belief Model variables, and outcomes of the study. At the same time, the qualitative part indicates what participants say about their reasons, perceptions, concerns about safety, and the methods of decision-making. All together it demonstrates how demographic, psychological, experience, and information factors influence knowledge, attitudes, and behavior related to ashwagandha among Saudi adults.

## 4 Aim of the study

The aim of this mixed-methods study was to comprehensively explore ashwagandha use among Saudi adults. It examined knowledge, attitudes, safety perceptions, and usage patterns. The study also aimed to understand behavioral, psychological, and sociocultural factors influencing its use within the Health Belief Model (HBM) framework.

### 4.1 Specific objectives

1. To assess Saudi adults’ knowledge regarding the benefits, risks, dosage, and safety considerations associated with ashwagandha use.
2. To examine attitudes, beliefs, and safety perceptions related to ashwagandha among Saudi adults.
3. To identify patterns, motivations, and sociodemographic predictors associated with ashwagandha use.
4. To examine participants’ lived experiences, decision-making processes, perceived benefits and barriers, and self-regulation practices related to ashwagandha use, using qualitative interviews.
5. To integrate quantitative and qualitative findings to develop a comprehensive understanding of ashwagandha-related health behaviors among Saudi adults within the context of the Health Belief Model.

### 4.2 Research questions

- What specific knowledge do Saudi adults have regarding the benefits, risks, and safety of ashwagandha?
- How is ashwagandha viewed in terms of its effectiveness and safety
- What behavioral, psychological, and sociocultural factors influence ashwagandha use among Saudi adults?
- Are there demographic differences in knowledge, attitudes, and practices regarding ashwagandha use?
- What experiences do Saudi adult users report regarding ashwagandha’s effectiveness and potential adverse effects?

## 5 Methodology

### 5.1 Study design

This study used a convergent mixed-methods design by integrating quantitative and qualitative approaches. It explored adults’ knowledge, attitudes, and experiences with ashwagandha use. Quantitative results were presented according to STROBE guidelines for observational studies (Hancı et al., 2023). The qualitative component followed the COREQ checklist for qualitative research reporting (Palinkas et al., 2015). Data from both methods were collected simultaneously and merged for analysis.

### 5.2 Quantitative component

Study Population: Adults aware of or using ashwagandha supplements were purposively sampled to ensure diversity in age, gender, and education. Each participant had a unique code for anonymity. The minimum sample size was determined using standard cross-sectional formulas (50% prevalence, 95% confidence, 5% margin of error). A total of 518 participants completed the survey. Given the exploratory nature and lack of a national registry, convenience and snowball sampling were also used. A total of 518 individuals responded to the survey. After data screening, 10 questionnaires were excluded because of incomplete responses or substantial missing data. The final analytical sample therefore comprised 508 participants. All descriptive and inferential analyses were conducted using the complete analytical sample of 508 participants.

Note on sampling: Purposive sampling identified individuals with relevant experience and diverse backgrounds in ashwagandha use. This approach, common in health and social research, aims to collect rich, information-dense data rather than statistical representativeness. While enhancing diversity of perspectives, it limits generalizability and may introduce selection bias.

#### 5.2.1 Inclusion criteria

- Adults from Saudi Arabia (aged 18 years and older).
- Participants must have some familiarity with ashwagandha (either through personal use or general awareness).
- Ability to comprehend and read Arabic, as the questionnaire was in Arabic.
- Willingness to provide informed consent to participate in the study.

#### 5.2.2 Exclusion criteria

- Individuals aged <18 years.
- Participants who were not familiar with ashwagandha (e.g., had never heard of or used it).
- Individuals with cognitive impairments or serious mental health conditions that may interfere with their understanding of the questionnaire.

### 5.3 Sample size calculation

The required sample size for the quantitative component was estimated using the finite-population sample-size formula:

A=Z^2p1−p

n=\frac NAE^ 2N−1+A

where (n) is the required sample size, (N) is the estimated target-population size, (Z) is the standard normal value corresponding to the selected confidence level, (p) is the expected population proportion, (E) is the acceptable margin of error, and (A) is an intermediate value calculated using the first equation and subsequently entered into the second equation. This notation was used consistently to avoid using the same symbol for different purposes.

For a 95% confidence level, (Z = 1.96). Because the expected population proportion was unknown, (p = 0.50) was used to produce the most conservative sample-size estimate. The desired margin of error was set at (E = 0.05).

For the qualitative component, 20 participants were purposively selected from the survey respondents to ensure variation in age, gender, and patterns of ashwagandha use. Data saturation was reached by the 18th interview, with two additional interviews conducted to confirm that no substantially new codes or themes emerged.

### 5.4 Data collection tool

Data was collected using a self-administered questionnaire that included the following sections:

The demographic section collected information on participants’ age, gender, education level, marital status, and prior ashwagandha use. It also included questions on how often they used ashwagandha, where they learned about it, any chronic health conditions, and use of other herbal supplements which defined as the use of one or more additional plant-based or herbal products during the same period as ashwagandha use. While, concurrent supplement use: use of non-herba l dietary or nutritional supplements, such as omega-3, vitamin B12, magnesium, or melatonin, during the same period as ashwagandha use.

#### 5.4.1 Ashwagandha knowledge scale

The ashwagandha Knowledge Scale (AKS) was developed by the principal investigator of the present study. Its items were generated following a comprehensive review of the literature on herbal medicine, complementary therapies, ashwagandha (*Withania somnifera*), supplement safety, and public-health considerations. The instrument was designed to assess Saudi adults’ knowledge of ashwagandha, including its potential mental and physical health benefits, safety profile, adverse effects, long-term use, supporting scientific evidence, and regulatory considerations.

The scale was initially developed in English and subsequently translated and culturally adapted into Arabic to enhance its clarity, relevance, and suitability for the target population. The AKS comprises 20 items with three response options: “True,” “False,” and “I do not know.” The items are organized into four domains: mental health benefits (items 1–4, 6, and 18), physical health benefits (items 5, 7–9, and 19), safety and adverse effects (items 10–14 and 20), and scientific evidence and regulation (items 15–17). Each correct response is assigned a score of 1, whereas incorrect and “I do not know” responses are assigned a score of 0. Total scores range from 0 to 20, with higher scores indicating greater knowledge of ashwagandha. Knowledge levels are categorized as low (0–7), moderate (8–14), or high (15–20).

An expert panel of specialists in pharmacology, complementary medicine, and public health reviewed the scale to confirm its content validity. They checked each item for clarity, relevance, completeness, and how well it represented the topic. The Content Validity Index (CVI) showed good agreement among experts. Face validity was assessed among Saudi adults in the target group, confirming that the items were clear, easy to read, and culturally appropriate. A summary of the psychometric properties of the Ashwagandha Knowledge Scale (AKS) and Ashwagandha Attitude Scale (AAS) is presented in Table 1.

**TABLE 1:** Summary of psychometric properties of the study instruments.

Instrument	Domains	Number of items	Response format	Validation methods	Reliability
Ashwagandha knowledge scale (AKS)	Mental health benefits, physical health benefits, safety and side effects, scientific evidence and regulation	20	True/False/I do not know	Expert review, CVI, face validity, pilot testing, EFA, CFA	Cronbach’s α = 0.84
Ashwagandha attitude scale (AAS)	Perceived benefits, safety concerns, trust in personal health decisions	10	5-Point likert scale	Expert review, CVI, face validity, pilot testing, EFA, CFA	Cronbach’s α = 0.87

Construct validity was checked using Exploratory Factor Analysis (EFA) and Confirmatory Factor Analysis (CFA). The Kaiser-Meyer-Olkin (KMO) value was above 0.70, and Bartlett’s Test of Sphericity was significant (p < 0.05), showing the data were suitable for factor analysis. EFA showed acceptable factor loadings (≥0.40) and grouped items as expected. CFA confirmed the four-factor structure, with good model fit indices: CFI and TLI above 0.90, RMSEA below 0.08, and SRMR below 0.08. The scale’s internal consistency was strong, with a Cronbach’s alpha of 0.84.

A pilot study: was conducted before the main data collection to assess whether the questionnaire was clear and easy to understand. Based on feedback, minor wording changes were made. The pilot data were not included in the final analysis.

#### 5.4.2 Ashwagandha attitude scale

The Ashwagandha Attitude Scale (AAS) was developed by the principal investigator of the current study. The scale items were generated following a comprehensive review of the literature on beliefs about herbal medicine, complementary and alternative medicine use, health-related attitudes, decision-making, and motivations for using dietary and herbal supplements. The instrument was designed to assess Saudi adults’ beliefs, perceptions, and attitudes toward ashwagandha (*W. somnifera*).

The AAS consists of 10 items rated on a five-point Likert scale ranging from 1, “strongly disagree,” to 5, “strongly agree.” The items cover three domains: perceived benefits of ashwagandha, perceived safety and risks, and trust and autonomy in health-related decision-making. Items 3 and 10 are negatively worded and are reverse-scored before calculating the total score. The total AAS score ranges from 10 to 50, with higher scores indicating more favorable attitudes toward ashwagandha. The scale was developed in English and subsequently translated and culturally adapted into Arabic to ensure clarity, relevance, and suitability for the Saudi adult population.

The instrument received expert evaluation and thorough testing to verify its validity, clarity and suitability for the culture. The factor analysis results indicate the establishment of acceptable factor-loadings and suitable values of the model-fit indices. In addition, the Kaiser–Meyer–Olkin index was more than 0.70 and Bartlett’s test of sphericity showed significance. Confirmatory factor analysis provided the evidence of the good model’s fit, with the values of CFI and TLI higher than 0.90 and the values of RMSEA and SRMR lower than 0.08. The instrument showed good internal consistency, with the alpha coefficient equal to 0.87.

Before conducting the main study, the research instrument was tested among Saudi adults to find out whether the items were clear and comprehensible. A few changes in wording were made according to participants’ observations, while the pilot results were excluded from the final analysis.

### 5.5 Data collection procedures

After getting approval from the Research Unit at KAIMRC and the Institutional Review Board (IRB), data were collected over 6 months. Participants received a structured questionnaire, available online through Google Forms and on paper. They filled out the questionnaire on their own, without the researchers’ help.

The study followed established reporting standards to ensure transparency, rigor, and reproducibility. For the quantitative part, the Strengthening the Reporting of Observational Studies in Epidemiology (STROBE) guidelines were used to guide the study design, variable selection, bias, analysis, and reporting. For the qualitative part, the Consolidated Criteria for Reporting Qualitative Research (COREQ) checklist guided reporting on team details, study design, participant selection, data collection, and analysis.

Qualitative data were collected through one-on-one, semi-structured interviews that were audio-recorded with participants’ consent. The recordings were transcribed exactly, checked for accuracy, and anonymized to protect confidentiality. A semi-structured guide was used to explore participants’ knowledge, views, experiences, and attitudes about ashwagandha. The guide included open-ended questions on information sources, perceived benefits and risks, cultural and social influences, and decision-making about using or not using ashwagandha. Each interview lasted about 30–45 min and was held in person or on a secure virtual platform, depending on the participant’s preference. Interviews took place in private, comfortable settings to encourage open discussion. The guide was divided into four phases to give structure to each session.

#### 5.5.1 Part I–introduction phase

At the beginning of each interview, the Part I: Introduction Phase. At the start of each interview, the study’s goals were explained. Participants were told about the importance of confidentiality and that joining was voluntary. Written or verbal consent was obtained for both participation and audio recording. Participants were asked general questions about their awareness of ashwagandha. They were also asked how they learned about it and why they used or did not use it. The interview explored initial beliefs and outside influences, including family, friends, and healthcare providers.

#### 5.5.2 Part II: exploration phase

This phase involved a detailed discussion of participants’ experiences, including how they used ashwagandha, for how long, perceived benefits like stress reduction and better sleep, and any side effects. It also covered safety concerns, interactions with other medications, consulting healthcare professionals, trust in herbal products, satisfaction with ashwagandha, and recommendations for others.

#### 5.5.3 Part III: closing phase

At the end of each interview, participants could share any extra comments or thoughts. They were thanked for their time and input.

### 5.6 Sampling and data saturation

For the qualitative phase, participants were chosen from the quantitative survey using purposive sampling to ensure a wide range of ages, genders, education levels, ashwagandha use frequency, and attitudes toward herbal supplements. This approach provided diverse perspectives and improved the quality of the qualitative data. Eligibility regarding age was confirmed during recruitment and informed consent. Although some interviewees chose not to disclose their exact age during the interview, all participants were confirmed to be aged 18 years or older and were therefore included in the qualitative sample.

A total of 20 participants were purposively recruited and interviewed individually using a semi-structured interview guide. Recruitment, data collection, and preliminary analysis were conducted concurrently, enabling the research team to monitor the emergence of new codes and themes throughout the process. Data saturation was reached by the 18th interview, and two additional interviews were conducted to confirm that no substantially new information, codes, or themes emerged in relation to the study objectives.

Semi-structured interviews were guided by questions based on the study’s goals and relevant research. With participant consent, interviews were audio-recorded and transcribed word-for-word before analysis. Thematic analysis was used, following Braun and Clarke’s six-step method. To ensure trustworthiness, the research team regularly discussed coding and theme development and kept an audit trail of decisions made during analysis.

### 5.7 Integration of quantitative and qualitative findings

The quantitative aspect of the study followed the STROBE methodology and the qualitative one was done in accordance with the COREQ guidelines for better methodological strictness and transparency. A combination of quantitative and qualitative methods was used with a convergence mixed methods technique and joint display analysis (Table 2) that made the comparison, crosschecking, and interpretation more systematic.

**TABLE 2:** Joint display integration of quantitative and qualitative findings.

Quantitative finding	Qualitative theme	Integration type	Meta-inference
Approximately 49% of participants demonstrated low knowledge scores regarding ashwagandha benefits, risks, dosage, and safety considerations	Participants described uncertainty, misconceptions, and fragmented understanding regarding the appropriate use and safety of ashwagandha	Convergent	Both datasets confirm substantial public knowledge gaps and limited evidence-based understanding of ashwagandha
Social media platforms represented the primary information source for approximately 65% of participants	Participants frequently relied on influencers, online testimonials, family members, and friends when making decisions about ashwagandha use	Convergent and complementary	Informal information ecosystems strongly shape public perceptions and decision-making regarding herbal supplement use
Participants with previous ashwagandha use demonstrated significantly more positive attitudes toward the supplement (p < 0.05)	Participants reported that perceived improvements in stress relief, sleep quality, and emotional wellbeing increased their trust in ashwagandha and encouraged repeated use	Complementary	Positive personal experiences reinforce favorable attitudes toward ashwagandha despite limited scientific knowledge regarding safety and efficacy
Moderate levels of concern regarding side effects and safety were reported quantitatively	Participants expressed fears related to dosage uncertainty, product quality, dependency, and possible interactions with medications or supplements	Convergent	Participants simultaneously perceive ashwagandha as beneficial yet potentially risky, reflecting ambivalent health behavior patterns
Ashwagandha use was commonly associated with stress relief and emotional wellbeing	Participants described using ashwagandha during periods of stress, anxiety, sleep disturbance, and psychological exhaustion	Convergent	Perceived psychological benefits and stress-related motivations are key drivers of ashwagandha use among Saudi adults
No statistically significant association was identified between educational level and knowledge scores	Awareness and understanding varied considerably among participants regardless of formal educational attainment	Divergent	Herbal supplement literacy may be influenced more strongly by cultural beliefs, experiential learning, and digital media exposure than by formal education
Quantitative findings suggested relatively low concurrent use of multiple supplements	Participants frequently described combining ashwagandha with vitamins, herbal remedies, or other supplements	Divergent	The discrepancy may indicate underreporting in structured surveys or differences in how participants interpret supplement-use behaviors
A substantial proportion of participants reported uncertainty regarding appropriate dosage and duration of use	Participants described self-adjusting dosages based on online advice, trial-and-error experiences, or recommendations from non-professional sources	Complementary	Lack of standardized guidance contributes to self-directed and potentially unsafe usage practices
Quantitative findings showed limited consultation with healthcare professionals before using ashwagandha	Participants reported perceiving herbal supplements as “natural” and therefore not requiring medical consultation	Complementary	Cultural perceptions of herbal remedies as inherently safe may reduce professional healthcare engagement regarding supplement use
Participants generally demonstrated favorable attitudes toward herbal remedies overall	Participants associated herbal products with traditional healing practices, cultural familiarity, and holistic wellbeing	Complementary	Cultural trust and traditional beliefs substantially influence acceptance and continued use of herbal supplements such as ashwagandha

With the help of integration, it has been found that both quantitative and qualitative results are in high conformity. Quantitative results showed that a majority of participants did not know much about the action of ashwagandha, effective dose, safety, contraindications, and side effects. The results of qualitative interviews support the quantitative findings as the participants admitted their doubts, wrong ideas, and incomplete knowledge frequently. Moreover, qualitative data show that the knowledge of participants is formed more thanks to personal experience and informal sources rather than scientific ones.

The numbers showed that prior ashwagandha consumption was significantly related to favorable views on the drug (p < 0.05). The qualitative findings helped explain this association. Participants who reported beneficial experiences with ashwagandha, including reduced stress, improved mood, greater relaxation, and enhanced wellbeing, generally expressed more favorable attitudes toward the supplement and a greater willingness to recommend it to others. These participants’ accounts provided contextual insight into the positive association between previous ashwagandha use and favorable attitudes toward its use.

As far as safety is concerned, the quantitative analysis showed moderate concern about side effects and prolonged usage. Interviews elaborated on these concerns revealing the issues like uncertainty about correct dosage, product quality, possibility of dependency, and interaction with medications or other supplements. All in all, the analysis indicates that respondents viewed ashwagandha as potentially beneficial, though not completely harmless.

Some divergence between the quantitative and qualitative findings was observed regarding concurrent supplement use. In this study, concurrent supplement use was defined as the use of one or more additional vitamins, minerals, herbal products, dietary supplements, or traditional remedies during the same period in which ashwagandha was used. The quantitative survey indicated that only a small proportion of participants reported using other products concurrently with ashwagandha. In contrast, the qualitative interviews indicated that concurrent use was more common than suggested by the survey responses.

This divergence may partly reflect differences in participants’ interpretations of the term “supplement use.” Some interviewees did not regard vitamins or traditional remedies as supplements, whereas others interpreted “concurrent use” as taking multiple products simultaneously rather than using them within the same general period. Interviewees reported using ashwagandha alongside vitamin D, iron, magnesium, omega-3 supplements, and other herbal products or traditional remedies. However, detailed information regarding the brand names, dosages, formulations, and complete ingredient compositions of the concurrently used products was not systematically collected. Therefore, the findings identify the general categories and examples reported by participants but do not allow a detailed assessment of individual ingredients or possible supplement–supplement interactions.

Although the quantitative analysis found no statistically significant association between educational level and ashwagandha knowledge scores, the qualitative findings indicated variation in participants’ awareness and information-seeking practices across educational backgrounds. Some participants with higher educational attainment reported consulting scientific or professional sources, whereas others, irrespective of educational level, relied on personal experiences, cultural beliefs, social relationships, and digital media. These findings suggest that formal education alone may not determine knowledge of ashwagandha; instead, awareness may be shaped by the interaction of experiential, cultural, social, and media-related influences.

The integrated findings lead to three major conclusions. First, knowledge of Ashwagandha among Saudi people turned out to be limited and acquired from personal experiences and informal sources rather than formal education or professional advice. Second, the integrated findings indicated that social networks and digital media played an important role in shaping participants’ attitudes and decisions regarding ashwagandha use. Family recommendations, peer experiences, online testimonials, and content shared through platforms such as TikTok and YouTube increased awareness and contributed to favorable perceptions of the supplement. Nevertheless, reliance on these informal sources may also have participants’ limited exposure to balanced, evidence-based information about potential adverse effects, contraindications, and medication interactions.

Finally, despite a generally favorable attitude toward ashwagandha and positive psychological outcomes of its use, the acquired knowledge about its use was still limited with regard to proper dosage, side effects, long-term safety, and interactions with medicine or other supplements. Thus, the results from the two types of research provided richer data for understanding people’s opinions, motivations, worries, and behavior as for the use of ashwagandha than if any of the researches were used separately.

### 5.8 Data management and analysis

Quantitative data were coded and entered into IBM SPSS Statistics, version 26. Before analysis, the dataset was screened and cleaned to identify missing values, data-entry errors, outliers, and inconsistencies. Qualitative transcripts, codes, and themes were organized and managed using NVivo 12 software. The quantitative and qualitative datasets were analyzed independently and subsequently integrated during the interpretation stage.

#### 5.8.1 Quantitative analysis

Descriptive statistics were used to summarize the quantitative data. Categorical variables were presented as frequencies and percentages. Continuous variables were summarized using means and standard deviations when approximately normally distributed and medians and interquartile ranges when their distributions were non-normal.

Composite scores were calculated for knowledge and safety perceptions and classified as low, moderate, or high according to the predefined scoring criteria. Patterns of ashwagandha use were assessed according to participants reported previous and current consumption. For each knowledge item, the percentage of correct responses was calculated by dividing the number of correct responses by the total number of valid responses to that item and multiplying the result by 100. Missing responses were excluded from the denominator for the corresponding item.

Associations between categorical variables were examined using the chi-square test. Fisher’s exact test was used when one or more expected cell frequencies were below five. Independent-samples t-tests and one-way analysis of variance were used to compare mean scores between two or more participant groups, respectively.

The assumptions required for parametric testing were evaluated before conducting group comparisons. Normality was assessed by examining the distributions of the continuous variables, including their skewness, histograms, and normal probability plots, whereas homogeneity of variance was evaluated across comparison groups. Parametric assumptions were considered unmet when variables showed substantial skewness, influential outliers, or unequal variances between groups. In these situations, the Mann–Whitney U test was used instead of the independent-samples t-test, and the Kruskal–Wallis’s test was used instead of one-way analysis of variance. Pearson’s correlation coefficient was used for approximately normally distributed continuous variables, whereas Spearman’s rank correlation coefficient was used for ordinal variables or continuous variables with non-normal distributions.

Multivariable regression analyses were conducted to identify factors associated with ashwagandha use, knowledge, and safety perceptions. Variables with a p value of <0.20 in the univariate analyses were considered for inclusion in the multivariable models. Binary logistic regression was used to identify predictors of ashwagandha use, whereas multiple linear regression was used to examine predictors of knowledge and safety-perception scores. The assumptions of the regression models, including linearity, multicollinearity, independence of errors, homoscedasticity, and the distribution of residuals, were evaluated as applicable. Adjusted odds ratios or regression coefficients were reported with their corresponding 95% confidence intervals. Statistical significance was established at p < 0.05.

#### 5.8.2 Qualitative analysis

Qualitative data were analyzed using thematic analysis. The analysis involved familiarization with the data, generation of initial codes, development of preliminary themes, review and refinement of the themes, definition and naming of the final themes, and preparation of the analytical narrative. The transcripts were reviewed repeatedly to ensure that the themes accurately reflected participants’ accounts.

The researchers compared and discussed the codes and emerging themes to enhance the consistency and credibility of the analysis. An audit trail documenting coding decisions and theme development was maintained throughout the analytical process. Reflexive notes and thick descriptions of participants’ experiences and the study context were also used to strengthen the trustworthiness and transparency of the findings.

#### 5.8.3 Mixed-methods integration

The quantitative and qualitative datasets were analyzed independently and subsequently integrated during the interpretation stage using a convergent parallel approach. Integration was achieved through methodological triangulation, comparison of the findings, and the development of joint displays. Areas of convergence, complementarity, and divergence between the two datasets were examined. The qualitative findings were used to explain and contextualize the quantitative results, particularly those concerning participants’ motivations, beliefs, information sources, patterns of use, and safety concerns related to ashwagandha.

#### 5.8.4 Quantitative results

Table 3 presents the demographic and health-related characteristics of the 508 participants included in the final analysis. Of the original 518 respondents, 10 were excluded because their questionnaires were incomplete or contained substantial missing data. Participants were, 35.6% aged below 20 years and 44.1% aged 20–29 years. Females constituted most of the sample (78.9%). Regarding educational attainment, the two largest groups were participants holding a bachelor’s degree (59.3%) and high school graduates (26.6%); the remaining participants were distributed across other educational categories. Most participants were single (80.5%).

**TABLE 3:** Distribution of the demographic characteristics of the study sample, N = 508.

​	n	%
Age (years)		
<20	181	35.6
20–29	224	44.1
30–39	45	8.9
40–49	43	8.5
50 or more	15	3.0
Gender		
Male	107	21.1
Female	401	78.9
Level of education		
Less than high school	4	0.8
High school graduate	135	26.6
Diploma	25	4.9
Bachelor’s degree	301	59.3
Master’s degree	23	4.5
Doctoral degree	20	3.9
Marital status		
Single	409	80.5
Married	89	17.5
Divorced	10	2.0
Previous use of ashwagandha		
No	383	75.4
Yes	125	24.6
Frequency of ashwagandha use		
Not at all	378	74.4
Rarely	27	5.3
Occasionally	24	4.7
Monthly	48	9.4
Weekly	11	2.2
Daily	20	3.9
Presence of any chronic health conditions		
No	459	90.4
Yes	49	9.6
If yes (n = 49)		
Heart disease	9	18.4
Diabetes	6	12.2
Hypothyroidism	10	20.4
Chronic fatigue	3	6.1
Arthritis (rheumatism)	7	14.3
Blood pressure	7	14.3
Psoriasis	7	14.3
Previous diagnosis with anxiety or any mental disorders		
No	430	84.6
Yes	78	15.4
If yes (n = 78)		
Chronic anxiety	11	14.1
ADHD	15	19.2
Depression	8	10.3
Tension	5	6.4
Panic and anxiety attacks	11	14.1
Post-traumatic stress disorder	14	17.9
Obsessive-compulsive disorder	14	17.9
Concurrent use of other herbal supplements		
No	470	92.5
Yes	38	7.5
If yes (n = 38)		
Turmeric	9	23.7
Cinnamon	10	26.3
Moringa	9	23.7
Spearmint tea	10	26.3

Regarding previous use, 125 participants, representing 24.6% of the total sample, reported having used ashwagandha, whereas 383 participants (75.4%) reported no previous use. Current frequency of use was assessed separately. Of the total sample, 378 participants (74.4%) selected “not at all,” whereas 130 participants (25.6%) reported using ashwagandha at varying frequencies: rarely (5.3%), occasionally (4.7%), monthly (9.4%), weekly (2.2%), or daily (3.9%).

Previous ashwagandha use was significantly associated with attitude level (p < 0.001). When percentages were calculated within each attitude category, previous users accounted for 57.4% of participants with a positive attitude, compared with 12.3% of those with a negative attitude. These subgroup percentages should be distinguished from the overall prevalence of previous use in the full sample, which was 24.6%.

Most participants reported no chronic health conditions (90.4%) and no prior diagnosis of anxiety or other mental disorders (84.6%). The use of other herbal supplements was uncommon (7.5%), with cinnamon (26.3%) and spearmint tea (26.3%) being the most frequently reported among users.

Figure 2 illustrates participants’ sources of information about ashwagandha. Social media was the most frequently reported source (45.3%), followed by herbal shops or pharmacies (27.0%) as reported by participants. Friends and family were identified as a source of information by 14.4% of participants, whereas 9.8% reported obtaining information from internet articles. Healthcare professionals were the least frequently reported source, cited by only 3.5% of participants. These findings describe where participants obtained information and should not be interpreted as evidence that they were willing to recommend ashwagandha to others.

**FIGURE 2:**
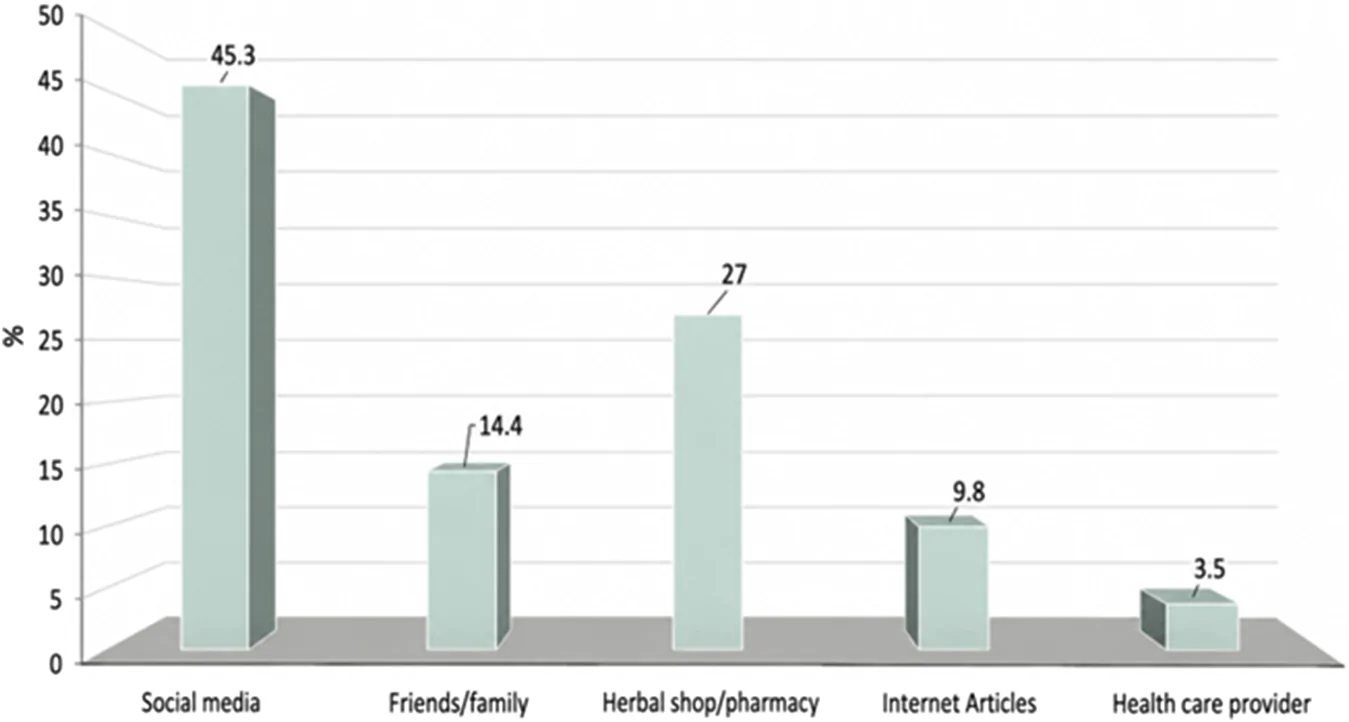
Primary source of information about ashwagandha.

The low endorsement of recommending ashwagandha to family or friends (effect size = 0.13) suggests that participants were hesitant to actively promote its use, even when some had previously received information about it through their social networks.

Table 4: There were significant variations in participants’ knowledge concerning ashwagandha across different categories with respect to the medicinal plant’s effect on mental health, physical health, safety issues, and regulation. It is evident from Table 3 that the percentage of correct answers ranged from 17.7% to 71.9%, thus suggesting a significant variation in terms of knowledge. Percentages were calculated using the number of correct responses for each item divided by the number of valid responses to that item.

**TABLE 4:** Distribution of the ashwagandha knowledge Scale with 95% CI and effect size (N = 508).

Item	Incorrect n (%)	Correct n (%)	95% CI (correct %)	Cohen’s h
Mental health benefits				
Adaptogen (stress coping)	143 (28.1)	365 (71.9)	67.9–75.7	0.46
Reduces cortisol	166 (32.7)	342 (67.3)	63.2–71.2	0.36
Enhances memory	283 (55.7)	225 (44.3)	40.1–48.6	0.11
Improves sleep	157 (30.9)	351 (69.1)	65.0–73.0	0.40
Reduces anxiety/depression	191 (37.6)	317 (62.4)	58.1–66.5	0.25
Neurodegenerative diseases	385 (75.8)	123 (24.2)	20.7–28.0	0.53
Physical health benefits				
Anti-inflammatory/antioxidant	280 (55.1)	228 (44.9)	40.6–49.1	0.10
Muscle strength/endurance	325 (64.0)	183 (36.0)	31.9–40.3	0.28
Blood sugar control	337 (66.3)	171 (33.7)	29.7–37.9	0.34
Male reproductive health	353 (69.5)	155 (30.5)	26.6–34.7	0.39
Reduces fatigue	348 (68.5)	160 (31.5)	27.6–35.6	0.37
Safety and side effects				
Completely risk-free (incorrect)	395 (77.8)	113 (22.2)	18.8–26.1	0.58
GI side effects	322 (63.4)	186 (36.6)	32.5–40.8	0.27
Autoimmune caution	333 (65.6)	175 (34.4)	30.4–38.6	0.31
Safe in pregnancy/children (incorrect)	418 (82.3)	90 (17.7)	14.6–21.3	0.68
Liver enzyme changes	306 (60.2)	202 (39.8)	35.6–44.1	0.21
Thyroid caution	257 (50.6)	251 (49.4)	45.1–53.7	0.02
Scientific evidence and regulation				
Strong evidence for all claims (incorrect)	392 (77.2)	116 (22.8)	19.4–26.8	0.56
SFDA regulation	305 (60.0)	203 (40.0)	35.8–44.3	0.20
Safe with all medications (incorrect)	411 (80.9)	97 (19.1)	16.0–22.7	0.64

CI: Confidence Interval. Cohen’s h was calculated to estimate the effect size for deviation from a reference proportion of 0.50. Values of 0.20, 0.50, and 0.80 indicate small, medium, and large effect sizes, respectively.

High knowledge scores were recorded when it came to the benefits of ashwagandha on mental health. For instance, it is important to note that 71.9% (95% CI: 67.9–75.7; h = 0.46) of participants knew that ashwagandha functions as an adaptogen. Moreover, 69.1% (95% CI: 65.0–73.0; h = 0.40) of individuals correctly answered that ashwagandha helps improve sleep quality. However, it should be noted that there were fewer knowledgeable participants in terms of potential effects on neurodegenerative diseases (24.2%, 95% CI: 20.7–28.0; h = 0.53).

The participants demonstrated low knowledge concerning the benefits of ashwagandha on physical health. The correct answers varied between 30.5% and 44.9%. It appears that people are not fully aware of the positive impact of the medicinal plant on metabolism and reproduction, which includes its impact on blood sugar regulation (33.7%, h = 0.34) and the male reproductive system (30.5%)

Regarding safety and adverse effects, several knowledge gaps and misconceptions were identified. Most participants correctly rejected the statement that ashwagandha has no risks at all, with 77.8% providing the correct response (h = 0.58). However, substantial deficiencies remained regarding its safe use during pregnancy, for which only 17.7% responded correctly (h = 0.68), and its potential to cause drug interactions, for which 19.1% responded correctly (h = 0.64). Knowledge of thyroid-related precautions was nearly evenly divided, with 49.4% of participants providing the correct response (h = 0.02).

Regarding scientific evidence and regulation, a lack of knowledge prevailed as well. Only 40.0% were able to correctly identify the regulatory category of ashwagandha, while only 22.8% realized that not all claims about the health benefits of herbal supplements are well-supported by scientific evidence. In general, the results show that there was relatively small effect sizes compared to the reference rate (0.50) for most items, although some important misconceptions were associated with large deviations.

Figure 3 Heatmap showing the level of awareness about ashwagandha regarding its applications in mental health, physical health, safety, and side effects, and scientific evidence and regulations. Different shades indicate the percentage of right answers for each statement, where darker shades mean lower percentages (i.e., red color = 0–20% right answers) while lighter shades indicate higher percentages (i.e., green color = 81–100% right answers).

**FIGURE 3:**
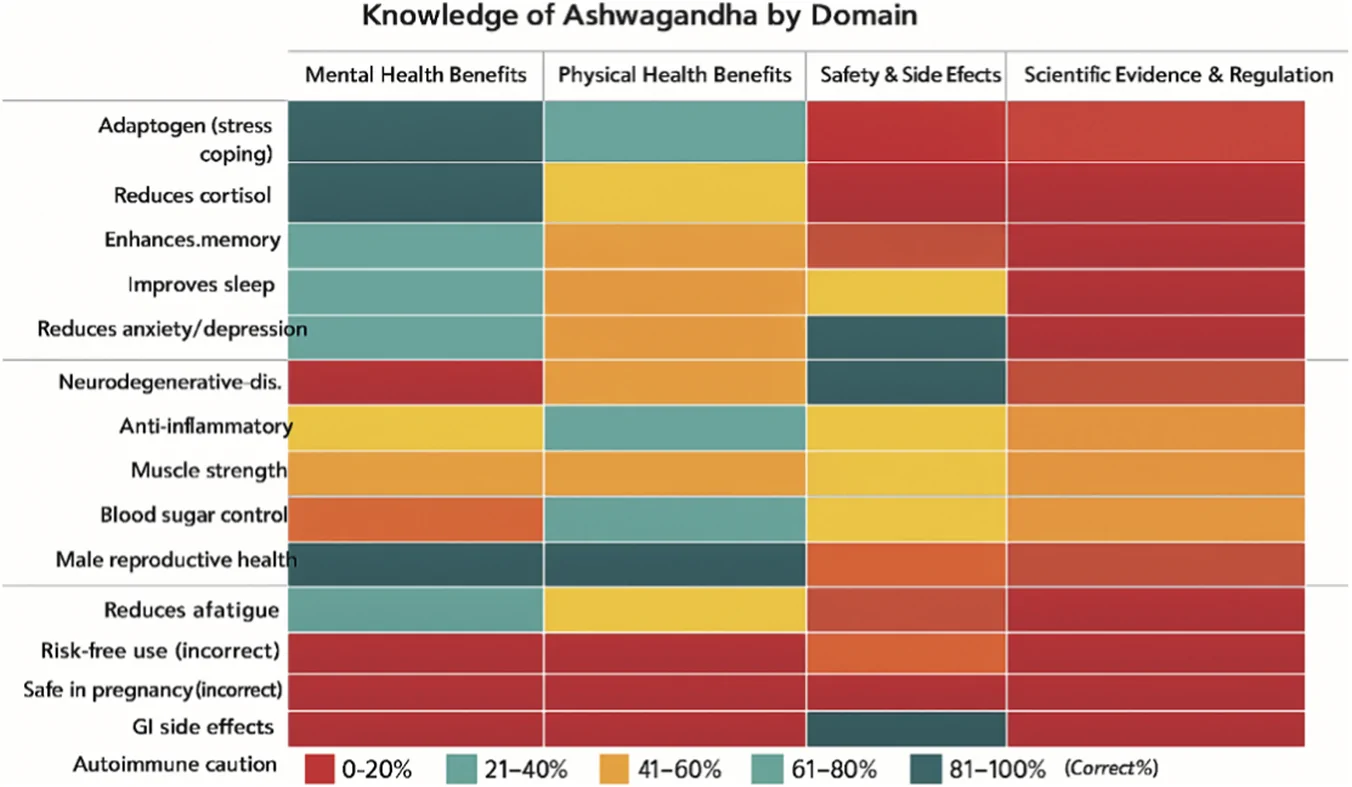
Heatmap of participants’ knowledge of ashwagandha by domain.

Figure 4 depicts the participants’ overall knowledge levels about ashwagandha. Almost half of the participants had low knowledge (49.4%, n = 251), while a similar percentage possessed moderate knowledge (47.2%, n = 240). In contrast, only a small fraction displayed high knowledge (3.3%, n = 17), suggesting that the study sample generally had limited awareness and understanding of ashwagandha.

**FIGURE 4:**
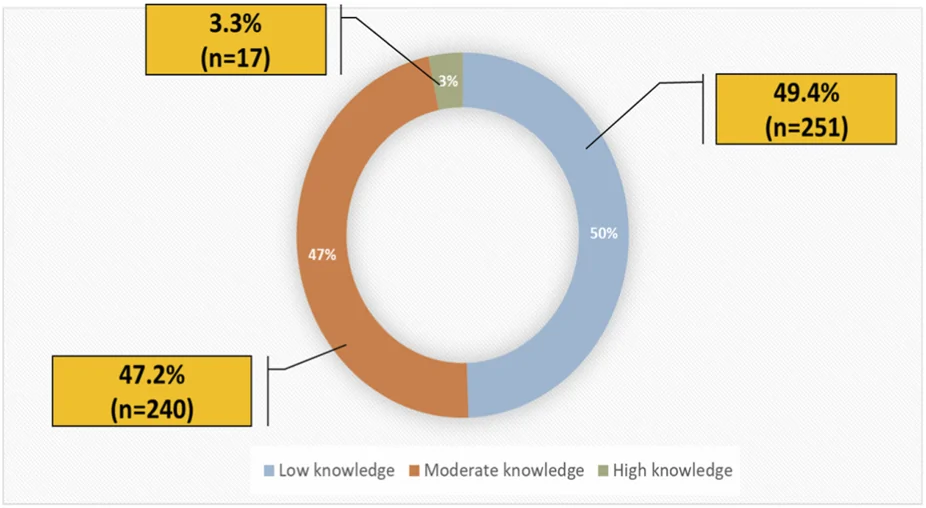
Distribution of knowledge scale levels of ashwagandha.

As shown in Table 5, participants’ attitudes toward ashwagandha demonstrated a complex pattern characterized by cautious endorsement and notable variability across individual items. The strongest effects were observed for the perceived need for increased public awareness (effect size = 0.45) and the belief that healthcare professionals should be consulted regarding herbal supplement use (effect size = 0.51). These findings indicate that participants recognized the importance of professional guidance and public education. However, this attitudinal preference should be distinguished from their actual behavior, as relatively few participants reported consulting healthcare professionals or relying on them as a primary source of information.

**TABLE 5:** Distribution of the ashwagandha attitude Scale with effect size (N = 508).

Item	Strongly disagree %	Disagree %	Neutral %	Agree %	Strongly agree %	Effect size (top-box %)
I Believe ashwagandha is effective in promoting general health	14.6	13.4	40.7	17.5	13.8	0.14
I Would use ashwagandha to reduce stress or anxiety	24.4	9.6	23.4	18.7	23.8	0.24
I Am concerned about the possible side effects of ashwagandha	15.0	9.4	26.6	18.1	30.9	0.31
I Trust herbal supplements more than pharmaceutical drugs	31.5	15.6	27.0	10.6	15.4	0.15
I Believe ashwagandha should be recommended by healthcare providers	15.9	12.2	28.0	14.6	29.3	0.29
I Would recommend ashwagandha to family or friends	31.7	17.7	25.8	11.4	13.4	0.13
I Think more awareness is needed about ashwagandha in Saudi Arabia	13.6	5.9	19.7	16.1	44.7	0.45
I Feel confident in deciding when and how to use ashwagandha	27.8	16.1	26.6	12.4	17.1	0.17
Herbal supplements like ashwagandha are a valuable part of healthcare	20.1	16.1	34.6	14.4	14.8	0.15
I Prefer to consult a healthcare professional before using herbal remedies	12.4	6.7	17.7	11.8	51.4	0.51

Effect size was estimated using the top-box approach (proportion of respondents selecting “Strongly Agree”), expressed as a decimal value. Interpretation thresholds were applied as follows: small effect (<0.20), moderate effect (0.20–0.40), and large effect (>0.40), reflecting the practical significance of attitudinal endorsement.

Moderate effects were identified for willingness to use ashwagandha for stress and anxiety (effect size = 0.24), concern about potential adverse effects (effect size = 0.31), and support for using ashwagandha when recommended by a healthcare provider (effect size = 0.29). Collectively, these findings suggest that participants acknowledged the value of professional advice and perceived potential benefits of ashwagandha, while remaining concerned about its safety. Nevertheless, recognition of the importance of healthcare-provider guidance did not necessarily translate into actual consultation practices.

In contrast, relatively small effect sizes were observed for perceived general health benefits (effect size = 0.14), confidence in personal decision-making (effect size = 0.17), and trust in herbal supplements over pharmaceutical medications (effect size = 0.15). Additionally, low endorsement of recommending ashwagandha to family or friends (effect size = 0.13) suggests hesitancy in actively promoting its use. Collectively, these findings indicate limited confidence and cautious attitudes toward independent use.

Figure 5 depicts participants’ opinions about ashwagandha use, most respondents, 62.0%, maintain a neutral position, showing neither positive nor negative feelings. This indicates a general sense of indifference or absence of strong opinions within the group. On the other hand, 28.7% of participants expressed negative attitudes toward ashwagandha, reflecting concerns about its safety, effectiveness, or unsupervised use. This proportion highlights the persistence of reservations among a substantial subgroup of participants despite the generally favorable or neutral perceptions observed in the overall sample. Conversely, only a small fraction, 9.3%, display a positive attitude, suggesting an opportunity for improvement or engagement to enhance perceptions.

**FIGURE 5:**
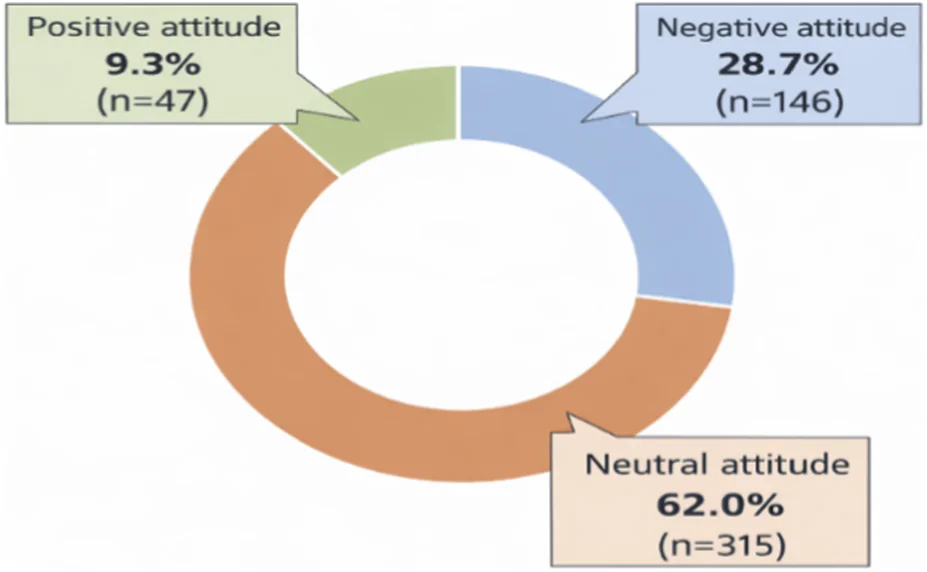
Distribution of the attitude scale level of ashwagandha, Data presented in Table 6 indicates that both demographic features and health-related factors of participants had an effect on their ashwagandha knowledge levels. The variable of age showed no significance in knowledge levels (p = 0.441). Even though participants in the age category of 20–29 years obtained 52.9% of the high knowledge level. Regarding age, participants aged 20–29 years constituted the largest proportion of the [low/moderate/high]-knowledge group. These percentages describe the age distribution within each knowledge-level category and should not be interpreted as the proportion of all participants aged 20–29 years who achieved that particular knowledge level.

**TABLE 6:** Association between the demographic characteristics of the study sample and knowledge level N-508.

Demographic characteristics	Low knowledge		Moderate knowledge		High knowledge		Chi – square/Fisher’s exact test	
	n	%	n	%	n	%	X2	P
Age (Years)								
<20	83	33.1	94	39.2	4	23.5	7.925	0.441
20–29	114	45.4	101	42.1	9	52.9		
30–39	21	8.4	23	9.6	1	5.9		
40–49	22	8.8	19	7.9	2	11.8		
50 or more	11	4.4	3	1.3	1	5.9		
Gender								
Male	49	19.5	49	20.4	9	52.9	10.809	0.004^*^
Female	202	80.5	191	79.6	8	47.1		
Level of education								
Less than high school	1	0.4	3	1.3	0	0.0	10.916	0.364
High school graduate	75	29.9	57	23.8	3	17.6		
Diploma	6	2.4	18	7.5	1	5.9		
Bachelor’s degree	146	58.2	144	60.0	11	64.7		
Master’s degree	12	4.8	10	4.2	1	5.9		
Doctoral degree	11	4.4	8	3.3	1	5.9		
Marital status								
Single	205	81.7	190	79.2	14	82.4	2.432	0.657
Married	41	16.3	46	19.2	2	11.8		
Divorced	5	2.0	4	1.7	1	5.9		
Previous use of ashwagandha								
No	196	78.1	175	72.9	12	70.6	1.987	0.370
Yes	55	21.9	65	27.1	5	29.4		
Frequency of ashwagandha use								
Not at all	196	78.1	171	71.3	11	64.7	23.553	0.009^*^
Rarely	11	4.4	13	5.4	3	17.6		
Occasionally	15	6.0	9	3.8	0	0.0		
Monthly	20	8.0	28	11.7	0	0.0		
Weekly	2	0.8	7	2.9	2	11.8		
Daily	7	2.8	12	5.0	1	5.9		
Presence of any chronic health conditions								
No	226	90.0	217	90.4	16	94.1	0.306	0.858
Yes	25	10.0	23	9.6	1	5.9		
Previous diagnosis with anxiety or any mental disorders								
No	206	82.1	208	86.7	16	94.1	3.207	0.201
Yes	45	17.9	32	13.3	1	5.9		
Concurrent use of other herbal supplements								
No	228	90.8	225	93.8	17	100.0	2.927	0.231
Yes	23	9.2	15	6.3	0	0.0		

*Statistically significant at p < 0.05.

Knowledge level was significantly associated with gender (p = 0.004). Among the 17 participants classified as having high knowledge, 9 (52.9%) were male and 8 (47.1%) were female. In contrast, among the 251 participants classified as having low knowledge, 49 (19.5%) were male and 202 (80.5%) were female. These percentages describe the gender composition within each knowledge-level category. They do not indicate that 52.9% of all male participants had high knowledge or that 80.5% of all female participants had low knowledge.

Educational level was not significantly associated with knowledge level (p = 0.100). Although participants with a bachelor’s degree constituted the largest proportion of the sample and of several knowledge categories, this finding likely reflects their overrepresentation in the study population rather than an independent effect of educational attainment on ashwagandha knowledge. Therefore, no conclusion can be drawn that participants with a bachelor’s degree had better knowledge than those with other educational backgrounds.

Analyses revealed that education was not significantly associated with knowledge level (p = 0.364). Among participants in the high-knowledge group, 64.7% had a bachelor’s degree, although this possibly reflects the overrepresentation of this group in the total sample. Furthermore, knowledge level was not significantly associated with marital status (p = 0.657) or previous ashwagandha use (p = 0.370). Ashwagandha usage frequency was found to be related to knowledge level (p = 0.009). Among the 17 participants classified as having high knowledge, 11 (64.7%) selected “not at all” for frequency of ashwagandha use, 2 (11.8%) reported weekly use, and 1 (5.9%) reported daily use. However, these figures refer to the high-knowledge group and do not prove that frequent users knew more than non-users. Chronic health conditions, previous diagnoses of anxiety or other mental disorders, and Concurrent use of other herbal supplements were not significantly associated with knowledge level (all (p > 0.05).

The relationship between participants’ knowledge of ashwagandha and their attitudes toward its use is presented in Figure 6. A statistically significant positive correlation was observed between knowledge and attitude scores (r = 0.176), (p < 0.001), indicating that higher knowledge scores were associated with slightly more favorable attitudes. However, the small correlation coefficient and the gentle upward slope of the regression line indicate that the strength of this relationship was weak.

**FIGURE 6:**
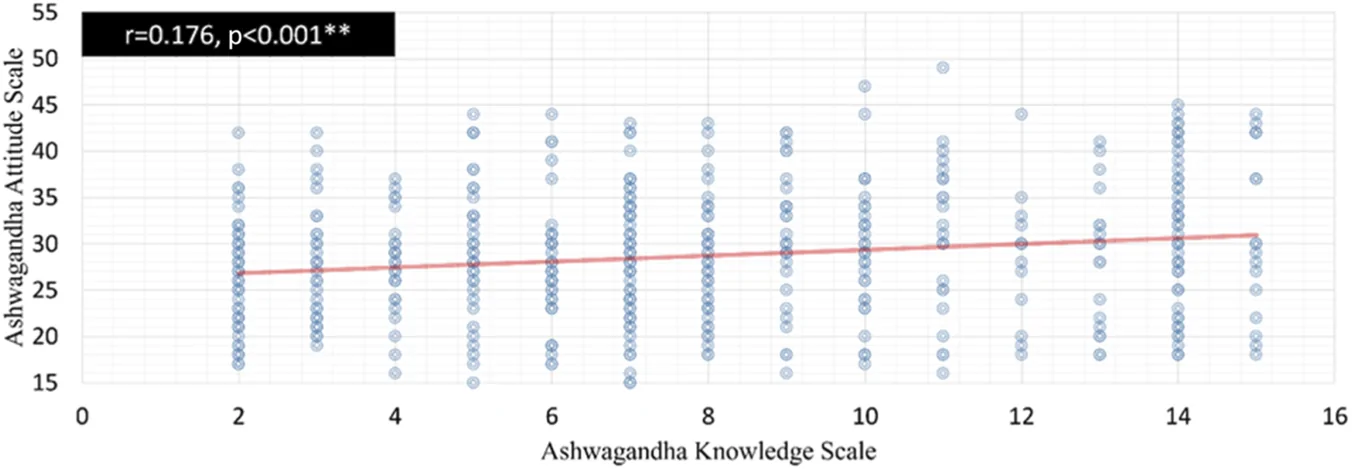
Correlation between ashwagandha knowledge scale and ashwagandha attitude scale.

Considerable variation in attitude scores was observed among participants with similar levels of knowledge, suggesting that knowledge alone did not adequately explain attitudes toward ashwagandha. Other factors may also have contributed to participants’ attitudes, including previous experience with ashwagandha, perceived benefits following its use, cultural beliefs, and the sources from which information was obtained. This interpretation was supported by the qualitative findings, in which participants with favorable personal experiences frequently expressed more positive attitudes, whereas reliance on family recommendations, social media, or other informal information sources also shaped perceptions of the benefits and safety of ashwagandha.

Table 7 presents the associations between participants’ demographic and health-related characteristics and their attitudes toward ashwagandha. Age was significantly associated with attitude level (p = 0.036). Participants aged 20–29 years constituted 48.9% of the positive-attitude category. However, because this percentage represents the age distribution within the positive-attitude group, it should not be interpreted as indicating that 48.9% of all participants aged 20–29 years had positive attitudes. No specific differences between individual age groups were inferred unless supported by *post hoc* comparisons.

**TABLE 7:** Association between the demographic characteristics of the study sample and attitude level, N = 508.

Demographic characteristics	Negative attitude		Neutral attitude		Positive attitude		Chi – square/Fisher’s exact test	
	n	%	n	%	n	%	X^2^	P
Age (years)								
<20	43	29.5	123	39.0	15	31.9	16.509	0.036*
20–29	61	41.8	140	44.4	23	48.9		
30–39	19	13.0	22	7.0	4	8.5		
40–49	20	13.7	21	6.7	2	4.3		
50 or more	3	2.1	9	2.9	3	6.4		
Gender								
Male	21	14.4	73	23.2	13	27.7	5.993	0.050^*^
Female	125	85.6	242	76.8	34	72.3		
Level of education								
Less than high school	0	0.0	2	0.6	2	4.3	15.980	0.100
High school graduate	39	26.7	87	27.6	9	19.1		
Diploma	11	7.5	12	3.8	2	4.3		
Bachelor’s degree	84	57.5	188	59.7	29	61.7		
Master’s degree	9	6.2	12	3.8	2	4.3		
Doctoral degree	3	2.1	14	4.4	3	6.4		
Marital status								
Single	111	76.0	260	82.5	38	80.9	4.580	0.333
Married	30	20.5	50	15.9	9	19.1		
Divorced	5	3.4	5	1.6	0	0.0		
Previous use of ashwagandha								
No	128	87.7	235	74.6	20	42.6	39.293	<0.001**
Yes	18	12.3	80	25.4	27	57.4		
Frequency of ashwagandha use								
Not at all	125	85.6	234	74.3	19	40.4	62.664	<0.001**
Rarely	6	4.1	14	4.4	7	14.9		
Occasionally	2	1.4	20	6.3	2	4.3		
Monthly	10	6.8	31	9.8	7	14.9		
Weekly	3	2.1	4	1.3	4	8.5		
Daily	0	0.0	12	3.8	8	17.0		
Presence of any chronic health conditions								
No	131	89.7	286	90.8	42	89.4	0.189	0.910
Yes	15	10.3	29	9.2	5	10.6		
Previous diagnosis with anxiety or any mental disorders								
No	116	79.5	275	87.3	39	83.0	4.840	0.089
Yes	30	20.5	40	12.7	8	17.0		
Concurrent use of other herbal supplements								
No	138	94.5	290	92.1	42	89.4	1.617	0.446
Yes	8	5.5	25	7.9	5	10.6		

*p < 0.05; **p < 0.01.

The association between gender and attitude level was at the threshold of statistical significance (p = 0.050). Females constituted 72.3% of the positive-attitude category, whereas males constituted 27.7%. These percentages reflect the gender composition of the positive-attitude group rather than the proportion of females and males who developed positive attitudes. Given that females represented 78.9% of the total sample, this finding should be interpreted cautiously and does not independently demonstrate that females were more likely than males to have positive attitudes.

Educational level was not significantly associated with attitude level (p = 0.100). Participants with a bachelor’s degree constituted approximately 60% of each attitude category, which was consistent with their predominance in the overall sample. Marital status was also not significantly associated with attitudes toward ashwagandha (p = 0.333).

Previous ashwagandha use was significantly associated with attitude level (p < 0.001). Previous users constituted 57.4% of the positive-attitude category, compared with 12.3% of the negative-attitude category. This pattern suggests that previous experience with ashwagandha was associated with more favorable attitudes, although the cross-sectional design does not establish whether use resulted in positive attitudes or whether favorable attitudes encouraged use.

Frequency of ashwagandha use was also significantly associated with attitude level (p < 0.001). Among participants in the positive-attitude category, 17.0% reported daily ashwagandha use, whereas no participants in the negative-attitude category reported daily use. This finding indicates that daily use was observed only among participants with favorable attitudes in the present sample. However, the cross-sectional design does not establish whether favorable attitudes encouraged more frequent use or whether repeated use and perceived benefits contributed to more favorable attitudes.

Chronic health conditions, previous diagnoses of anxiety or other mental disorders, and Concurrent use of other herbal supplements were not significantly related to attitude level (all (p > 0.05). Therefore, no statistically significant evidence of a relationship between these factors and attitudes toward ashwagandha was identified in the present sample.

The results of the multiple linear regression analyses examining predictors of ashwagandha knowledge and attitude scores are summarized in Table 8. Previous ashwagandha use significantly predicted both knowledge scores (B = 1.194, SE = 0.538, t = 2.222, p = 0.027) and attitude scores (B = 2.841, SE = 1.248, t = 2.277, p = 0.023).

**TABLE 8:** Multiple linear regression analysis of sociodemographic and use-related predictors of ashwagandha knowledge and attitude scores (n = 508).

Predictor variables	Knowledge scale B (SE)	β	t	p-value	Attitude scale B (SE)	β	t	p-value
Constant	7.768 (1.727)	—	4.497	<0.001**	26.903 (2.945)	—	9.136	<0.001**
Age (years)	0.482 (0.257)	0.124	1.878	0.061	0.620 (0.437)	0.089	1.418	0.157
Gender	0.434 (0.462)	0.044	0.939	0.348	0.338 (0.787)	0.019	0.430	0.668
Educational level	0.024 (0.165)	0.007	0.144	0.885	0.165 (0.281)	0.027	0.585	0.559
Marital status	0.590 (0.736)	0.064	0.802	0.423	1.270 (0.918)	0.081	1.384	0.167
Previous use of ashwagandha	1.194 (0.538)	0.137	2.222	0.027*	2.841 (1.248)	0.171	2.277	0.023*
Frequency of ashwagandha use	0.234 (0.211)	0.089	1.108	0.268	0.798 (0.359)	0.168	2.225	0.027*
Presence of chronic health conditions	0.184 (0.630)	0.014	0.292	0.771	0.992 (1.073)	0.041	0.924	0.356
Previous diagnosis of anxiety or mental disorders	0.866 (0.508)	0.078	1.706	0.039*	2.447 (0.861)	0.123	2.843	0.005*
Concurrent use of other herbal supplements	0.665 (0.674)	0.044	0.987	0.324	2.064 (1.147)	0.076	1.799	0.073

Concurrent use of other herbal supplements: use of at least one additional herbal product during the same period as ashwagandha use. B, unstandardized regression coefficient; SE, standard error; \beta, standardized regression coefficient; CI, confidence interval.

* p < 0.05.

** p < 0.01.

***p < 0.001.

A previous diagnosis of anxiety or another mental health condition was more strongly associated with attitude scores (β = 0.12) than with knowledge scores (β = 0.08). However, its association with knowledge did not reach statistical significance (B = 0.866, SE = 0.508, t = 1.706, p = 0.089). Therefore, this variable should not be described as a significant predictor of knowledge. The corresponding coefficient and p value for the attitude model should be reported as presented in Table 8.

Frequency of ashwagandha use significantly predicted attitude scores (B = 0.798, SE = 0.359, t = 2.225, p = 0.027), but it was not a significant predictor of knowledge scores (p = 0.268). None of the sociodemographic variables, including age, gender, educational level, and marital status, significantly predicted either knowledge or attitude scores. Similarly, chronic health conditions and the Concurrent use of other herbal supplements were not significant predictors of either outcome. Concurrent herbal-supplement use was defined as the use of one or more additional herbal products during the same period in which ashwagandha was used. Pearson’s correlation analysis demonstrated a weak but statistically significant positive correlation between knowledge and attitude scores (r = 0.176, p < 0.001).

Figure 7 shows the forest plots which illustrate the standardized beta coefficients (β) and confidence intervals for predictors of ashwagandha attitude and knowledge scores. Prior use of ashwagandha and its frequency were the strongest positive predictors in both models. This indicates that individuals with prior experience and more frequent use demonstrated higher knowledge levels and more favorable attitudes toward ashwagandha.

**FIGURE 7:**
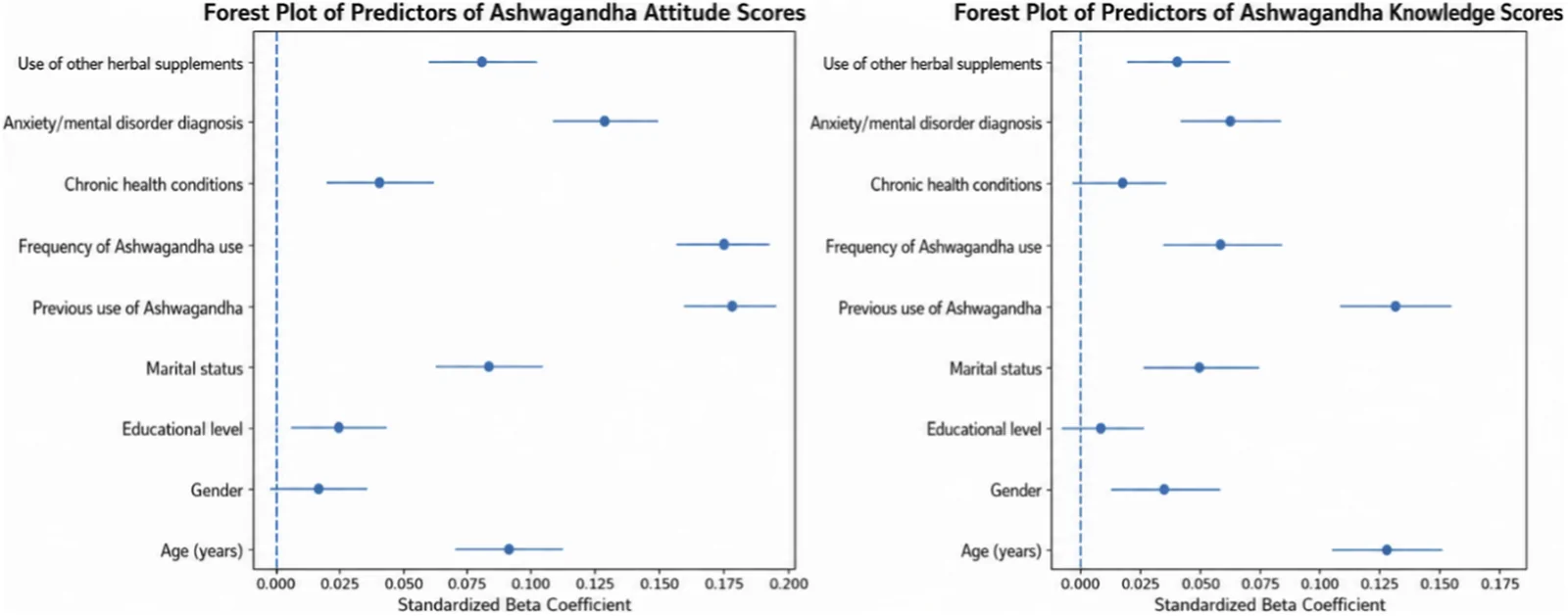
The predictors of knowledge and attitudes toward use of ashwagandha among participants.

When comparing the attitude and knowledge score models, prior ashwagandha use and frequency of use both showed the highest standardized beta coefficients in the attitude model (β = 0.17 for each). In the knowledge model, prior ashwagandha use was again one of the strongest predictors (β ≈ 0.14), but here age (β = 0.13) slightly surpassed frequency of use (β = 0.09). Anxiety/mental disorder diagnosis was more strongly associated with attitude scores (β = 0.12) than knowledge scores (β = 0.08). Meanwhile, marital status, use of other herbal supplements, chronic health conditions, educational level, and gender had moderate to weaker positive associations in both models, with the order and effect sizes varying slightly across the two outcomes.

In the knowledge-score model, previous ashwagandha use was a statistically significant positive predictor of knowledge. A previous diagnosis of anxiety or another mental disorder was also significantly associated with a higher knowledge score. Although age and frequency of ashwagandha use showed positive standardized coefficients, their associations were not statistically significant. Gender, educational level, marital status, chronic health conditions, and use of other herbal supplements also did not significantly predict knowledge scores.

Most predictors had confidence intervals above the null reference line, suggesting statistically meaningful positive associations. These findings suggest that previous exposure to ashwagandha and health-related experience, particularly a previous diagnosis of anxiety or another mental disorder, were associated with participants’ knowledge and attitudes. In contrast, the examined sociodemographic characteristics were not significant independent predictors.

#### 5.8.5 Qualitative results

Table 9 presents the demographic and relevant health-related characteristics of the qualitative participants (n = 20). The participants were predominantly young adults. Age data were available for 16 participants and ranged from 18–23 years–42 years or older, with a mean age of 26.7 ± 8.3 years; four participants (20%) did not report their age. Thirteen participants (65%) were male and seven (35%) were female, and all participants were Saudi nationals. Although they shared a broad national and sociocultural context, they were not assumed to be culturally identical, as their experiences and perspectives may have differed according to age, gender, education, marital status, socioeconomic circumstances, and personal experiences.

**TABLE 9:** Demographic characteristics, ashwagandha use, and concurrent herbal or nutritional supplement-use patterns of qualitative participants (n = 20).

Characteristic	Category/variable	n (%) or mean ± SD
Age (years)	18–23	6 (30%)
	24–35	4 (20%)
	36–41	3 (15%)
	≥42	3 (15%)
	Exact age not disclosed	4 (20%)
	Mean ± SD*	26.7 ± 8.3
Gender	Female	7 (35%)
	Male	13 (65%)
Nationality	Saudi	20 (100%)
Educational level/major	First-year university students: nursing, computer science, or health track	5 (25%)
	Bachelor’s degree in various disciplines	6 (30%)
	Doctoral degree, including biochemistry and cancer biology	2 (10%)
	Medical student/preclinical level	1 (5%)
	Other or not specified	6 (30%)
Marital status	Single	12 (60%)
	Married	2 (10%)
	Divorced	1 (5%)
	Not disclosed or unclear	5 (25%)
Monthly income (SAR)	<5,000	5 (25%)
	5,000–10,000	5 (25%)
	10,001–15,000	4 (20%)
	15,001–20,000	3 (15%)
	>20,000	2 (10%)
	Not disclosed	1 (5%)
	Mean ± SD^†^	10,850 ± 5,250
Concurrent use of herbal or nutritional supplements	Magnesium	3 (15%)
	Omega-3/fish oil	3 (15%)
	Iron, vitamin D, or vitamin B12	4 (20%)
	Creatine or zinc	2 (10%)
Herbal products	Chamomile	8 (40%)
	Cinnamon	7 (30%)
	Spearmint	3 (15%)
Nutritional supplements	Biotin	2 (10%)
	No concurrent herbal or nutritional supplement use reported	6 (30%)
Ashwagandha use status	Previously used intermittently or for a short period	8 (40%)
	Currently using occasionally	2 (10%)
​	Never used	6 (30%)
	Use status unclear or not specified	4 (20%)

Participants could report more than one herbal or nutritional product; therefore, percentages within the concurrent-use section are not expected to total 100%. Percentages are calculated based on the total qualitative sample (n = 20). Participants could report concurrent use of more than one herbal product or nutritional supplement; therefore, frequencies and percentages within the concurrent-use categories are not mutually exclusive and do not sum to 100%. “No concurrent herbal or nutritional supplement use reported” represents participants who did not report using any such products during the relevant period. The mean age was calculated using participants for whom exact age was available, and the mean monthly income was calculated using available reported income data. Concurrent use refers to products used during the same period as ashwagandha. Herbal products included chamomile, cinnamon, and spearmint, whereas dietary or nutritional supplements included biotin, vitamins and minerals, omega-3, fatty acids, magnesium, vitamin B12, and melatonin. Biotin was classified as a dietary supplement, not an herbal product. Four participants did not provide sufficient information to determine their ashwagandha-use status.

*p < 0.05; ^†^Fisher’s exact test.

Participants’ educational backgrounds ranged from first-year university education to doctoral-level qualifications, which may have contributed to variation in their health knowledge and perceptions. Most participants were single (60%), although marital-status information was unavailable for several participants. Reported monthly income also varied, with a mean of SAR 10,850 ± 5,250 among those who provided this information, indicating socioeconomic diversity within the sample.

Concurrent use of herbal products and nutritional supplements was common. The most frequently reported herbal products were chamomile (40%), cinnamon (35%), and spearmint (15%). Among nutritional supplements, magnesium and omega-3/fish oil were each reported by 15% of participants, while iron, vitamin D, or vitamin B12 were reported by 20%. Biotin was reported by 10% of participants. Because participants could report more than one concurrently used product, these percentages are not mutually exclusive. Six participants (30%) reported no concurrent use of herbal or nutritional supplements.

Regarding ashwagandha-use status, six participants (30%) had never used ashwagandha, two (10%) were current occasional users, and eight (40%) had tried briefly and discontinued. For the remaining four participants (20%), ashwagandha-use status was unclear or not specified. The findings were therefore interpreted within the broader Saudi sociocultural context while acknowledging the demographic and experiential variation among individual participants.

#### 5.8.6 Qualitative codebook

The analysis showed a clear picture of what participants know about ashwagandha, how they use it, and how they decide to take it. Most said they get information from social media, family, or pharmacists. The main benefits they noticed were less stress, better sleep, and a more stable mood. Some participants demonstrated awareness of potential risks associated with ashwagandha use, including emotional numbness, concerns about dependency, fear of adverse effects, and uncertainty regarding its long-term safety. These concerns reflected participants’ perceptions and experiences and should not be interpreted as clinically established adverse effects. Participants mostly use ashwagandha when they feel stressed or under academic pressure. They take it in capsule, powder, or tea form, but their preferences and how often they use it vary. Most said stress and lifestyle were the main reasons for using ashwagandha, and they included it as part of their health and fitness routines. Most participants were careful with ashwagandha, managing their own use and sometimes checking with doctors about its safety and effectiveness. Many used it along with other supplements. Participants evaluated their experiences with ashwagandha by describing perceived changes in stress, relaxation, sleep, mood, and physical wellbeing, as well as any unwanted effects. These accounts were coded as perceived effectiveness and physical or psychological responses during the thematic analysis.” Illustrative participant quotations have also been retained within the relevant subtheme to demonstrate how this interpretation was derived from the qualitative data.

Theme 1: Knowledge and awareness of ashwagandha.

**Table:** 

Subtheme	Definition	Example codes	Illustrative quote
1.1 Sources of information	How participants first learned about ashwagandha	TikTok videos, relatives’ recommendations, pharmacists’ advice	“I first heard about it on TikTok … ”
1.2 Understanding of benefits	Participants perceived or known benefits of ashwagandha	Calming effects, sleep support, mood stabilization	“It just makes one calm down … ”
1.3 Awareness of risks	Participants’ knowledge, uncertainty, or concerns regarding possible adverse effects	Emotional numbness, dependency concerns, fear of adverse effects, long-term safety concerns	“People … felt numbness in their emotions.”

Theme 2: Patterns of ashwagandha and supplement use.

**Table:** 

Subtheme	Definition	Example codes	Illustrative quote
2.1 Frequency and duration	How often and for how long ashwagandha was used	Exam-related use, intermittent use, short-term trial	“Two or three times before a test.”
2.2 Forms of administration	The formulations and methods through which ashwagandha was consumed	Capsules, powders, teas	“One capsule, 450 mg … ”
2.3 Concurrent use of other supplements	Use of ashwagandha alongside vitamins, herbal products, sports supplements, or other remedies	Vitamin D, zinc, magnesium, creatine, iron, herbal remedies	“I take vitamin D monthly … ”

Theme 3: Motivations for use.

**Table:** 

Subtheme	Definition	Example codes	Illustrative quote
3.1 Stress relief and relaxation	Use of ashwagandha to manage stress, anxiety, tension, or emotional discomfort	Examination stress, relaxation, anxiety reduction	“I used it because of stress before exams.”
3.2 Lifestyle and wellness influence	Use influenced by fitness routines, lifestyle practices, or broader wellness goals	Workout supplements, health routines, perceived nutritional deficiencies	“I use magnesium and creatine during workouts.”

Theme 4: Decision-making, self-monitoring, and safety concerns.

**Table:** 

Subtheme	Definition	Example codes	Illustrative quote
4.1 Cautious approach	Self-regulated use arising from uncertainty or concern about potential harm	Limiting dosage, intermittent use, temporary discontinuation	“I did not use it daily … ”
4.2 Consultation with professionals	Seeking healthcare advice before or during ashwagandha use	General practitioner advice, pharmacist counseling, safety recommendations	“A general practitioner advised me … ”
4.3 Self-monitoring and perceived effectiveness	Personal evaluation of benefits, symptoms, adverse effects, and the need to continue or discontinue supplements	Tracking mood, sleep, energy, physical symptoms, and perceived effectiveness	“I noticed iron deficiency symptoms … ”

Figure 8 presents the four main themes and their associated subthemes identified from participants’ interviews. Each theme is represented by a distinct color, with the corresponding subthemes positioned beneath it to illustrate their conceptual relationship. The infographic provides a concise overview of participants’ experiences, perceptions, motivations, and safety concerns regarding ashwagandha use. The connections among the themes represent recurring relationships identified across participants’ narratives, such as the influence of informal information sources on perceived benefits and safety concerns and the subsequent effect of these perceptions on patterns of use. The figure illustrates the organization and interrelationships of the qualitative findings rather than the frequency, magnitude, or relative importance of individual themes.

**FIGURE 8:**
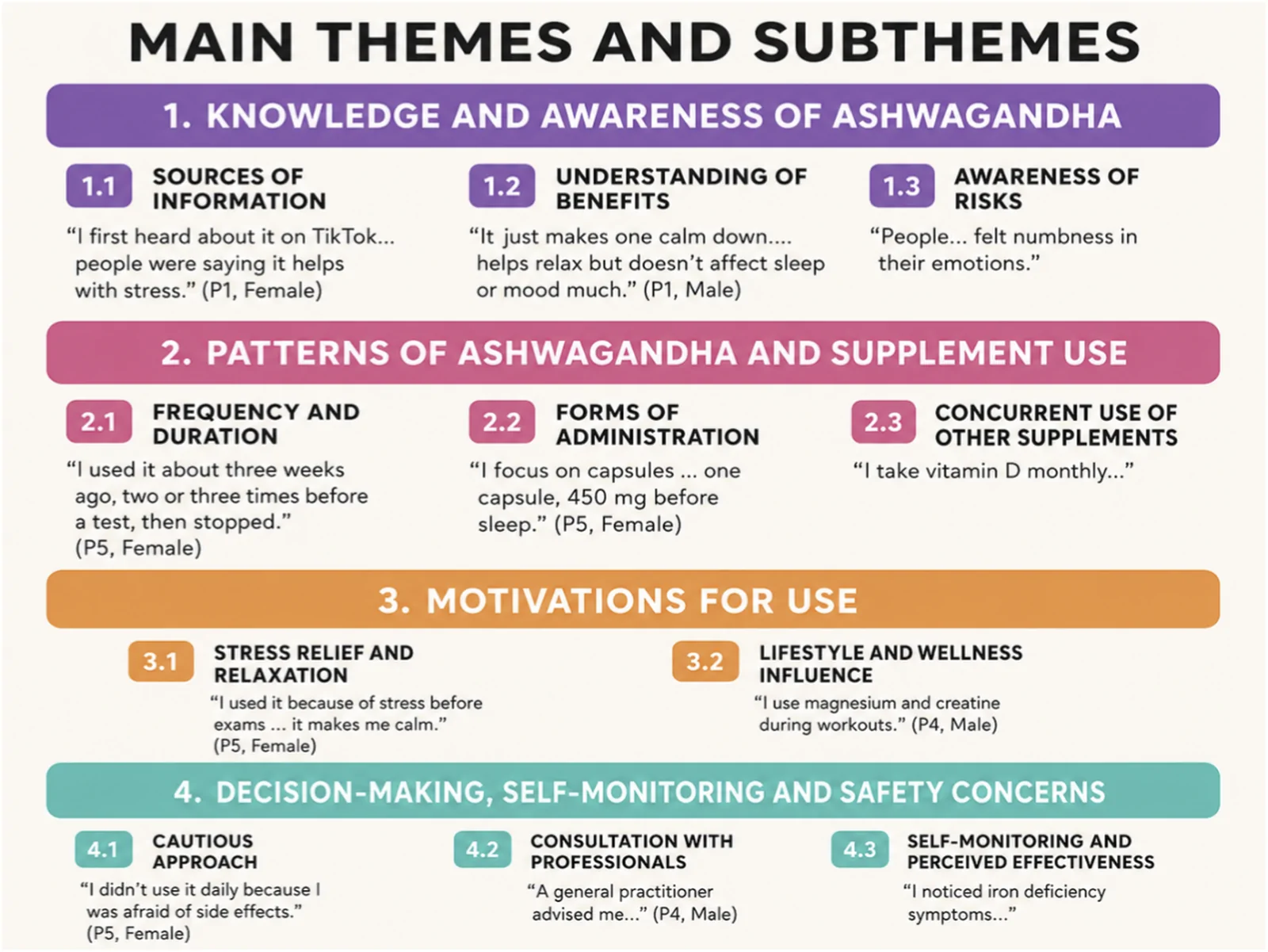
Summary of themes and subthemes in the qualitative data.

### 5.9 Narrative presentation of themes with embedded illustrative quotes

#### 5.9.1 Theme 1: knowledge and awareness of ashwagandha

Figure 8 summarizes the qualitative findings concerning participants’ sources of information, perceived benefits, and awareness of potential risks associated with ashwagandha. The interview data indicated that participants obtained information about ashwagandha from informal sources, including social media, family members, and friends. A smaller number of participants explicitly reported receiving information from professional sources, particularly pharmacists and physicians. Participants also expressed varying perceptions of the potential benefits and safety of ashwagandha.

##### 5.9.1.1 Sources of information

Several participants reported first learning about ashwagandha through informal information sources, particularly social media. TikTok was specifically identified as a source of information about its proposed stress-relieving effects. One participant stated:

“I first heard about it on TikTok … people were saying it helps with stress” (P1, 18, Female).

Family members and relatives also contributed to participants’ awareness and decisions regarding ashwagandha use. One participant explained:

“My relatives were taking it, and I asked about it. They said it alleviates tension and anxiety” (P5, 18, Female).

Some participants obtained information from healthcare professionals, including pharmacists and general practitioners. One participant reported:

“A pharmacist mentioned that it has both benefits and harms” (P2, 23, Female).

This account illustrates how consultation with a healthcare professional may provide information addressing both potential benefits and safety concerns.

##### 5.9.1.2 Perceived benefits

Participants described ashwagandha primarily in relation to calming, relaxation, stress reduction, and sleep. However, perceptions of its effectiveness differed across individual accounts. One participant described its perceived effect as limited:

“It just makes one calm down … helps relax but does not affect sleep or mood much” (P1, 35, Male).

Another participant referred to perceived benefits reported by relatives:

“According to my relatives, it alleviates tension and helps them sleep” (P5, 18, Female).

These accounts demonstrate variation in perceived effectiveness, ranging from general relaxation to reported improvements in tension and sleep.

##### 5.9.1.3 Awareness of potential risks

Some participants expressed concerns about the possible adverse effects and long-term safety of ashwagandha. These concerns were based mainly on personal perceptions, experiences, or information received from others rather than on professional clinical assessment. Fear of dependency and adverse effects influenced decisions to discontinue or avoid regular use. One participant stated:

“I stopped using it because I was afraid it could cause addiction or side effects” (P5, 18, Female).

Another participant described information received from others concerning emotional effects:

“People mentioned that over time they felt numbness in their emotions” (P6, 38, Female).

These accounts indicate that anecdotal information about dependency, emotional numbness, and other possible adverse effects contributed to cautious attitudes toward ashwagandha. Such statements represent participants’ perceptions and should not be interpreted as clinically established effects.

#### 5.9.2 Theme 2: patterns of ashwagandha and supplement use

##### 5.9.2.1 Frequency and duration

Considerable variation was observed in how frequently and for how long participants used ashwagandha. Some restricted their use to short periods of acute stress. One participant stated, “I used it about 3 weeks ago, two or three times before a test, then stopped” (P5, 18, Female). Others described sporadic use over a longer period: “I have been using it since 2022, but only about three or four times, whenever I feel stress” (P6, 38, Female). These accounts indicate that ashwagandha was generally used on an as-needed basis rather than as a regular daily supplement.

##### 5.9.2.2 Forms of administration

Capsules were the most commonly preferred formulation because of their convenience and perceived dosing precision. One participant stated, “I focus on capsules … one capsule, 450 mg before sleep” (P5, 18, Female), while another reported, “Capsules, yes … pure capsules” (P6, 38, Female). Participants expressed less interest in teas or powders, suggesting a preference for products that were easy to use and offered clearer dosing instructions. In addition, they described self-directed adjustments to the dosage and frequency of ashwagandha use. Reported daily doses of ashwagandha capsules varied, with some participants indicating that they took approximately 400 mg per day and others reporting daily doses of up to 700 mg. Some participants reported initially taking 400 mg per day for one week and subsequently increasing the dose to 700 mg per day after perceiving improvements in stress and sleep. “This information was based on participants’ self-reports and was not independently verified using product labels or clinical records.

Other participants reported using ashwagandha intermittently during examination periods or other periods of increased stress, discontinuing its use once the stressful situation or perceived stress had subsided.

##### 5.9.2.3 Concurrent use of other herbal and dietary supplements

Participants described incorporating ashwagandha into broader supplement routines that included vitamins, minerals, sports supplements, and products intended to support sleep or relaxation. Ashwagandha was primarily taken in capsule form, with reported daily doses of either 400 or 700 mg. Some participants used it intermittently during examination periods or other periods of increased stress and discontinued its use when the stressful situation or perceived stress had subsided. During the same general period, participants also reported using products such as vitamin D, vitamin B12, zinc, creatine, magnesium, omega-3 supplements, and melatonin. However, participants did not always indicate that these products were taken at exactly the same time as ashwagandha.

For example, one participant described a broader supplement routine: “I take vitamin D monthly, and zinc or creatine during workouts” (P4, 18, Male). Another stated, “Magnesium helps me relax and sleep better” (P5, 18, Female). These accounts illustrate participants’ broader supplement-use practices but should not be interpreted as direct evidence that all supplements were consumed simultaneously with ashwagandha.

#### 5.9.3 Theme 3: motivations for use

##### 5.9.3.1 Stress relief and relaxation

Stress reduction was the primary motivation for ashwagandha use, particularly during periods of academic pressure, work-related tension, or emotional strain. One participant reported, “I used it because of stress before exams … it makes me calm” (P5, 18, Female). Another explained, “It helps me feel relaxed and reduces tension” (P1, 35, Male). These accounts suggest that participants often experimented with ashwagandha as a self-directed strategy for managing stress and promoting relaxation.

##### 5.9.3.2 Lifestyle and wellness influence

For some participants, ashwagandha formed part of a broader wellness or fitness routine. It was used alongside supplements such as magnesium, creatine, vitamins, and iron. One physically active participant stated, “I use magnesium and creatine during workouts … helps with muscle intensity” (P4, 18, Male). Another participant explained, “I use vitamins and iron because of deficiency, but ashwagandha was for stress” (P2, 23, Female). These findings show that ashwagandha use was influenced not only by psychological needs but also by broader lifestyle and personal health practices.

#### 5.9.4 Theme 4: decision-making, self-monitoring, and safety concerns

##### 5.9.4.1 Cautious approach

Caution strongly influenced participants’ decisions regarding ashwagandha use. Concerns about side effects, dependency, and emotional changes led some participants to limit their dosage, discontinue use, or use the supplement only intermittently. One participant stated, “I did not use it daily because I was afraid of side effects” (P5, 18, Female). Another reported, “I stopped after 2 weeks, then took it again only when stressed” (P6, 38, Female). These patterns demonstrate a self-protective approach characterized by irregular and carefully monitored use.

##### 5.9.4.2 Consultation with professionals

Several participants sought professional advice before or during ashwagandha use. Medical guidance appeared to increase confidence, particularly when participants were experiencing stress-related symptoms. One participant stated, “A general practitioner advised me that my symptoms were stress-related, and suggested using it” (P5, 18, Female). Another explained, “A pharmacist explained the benefits and possible harms, especially for those with chronic diseases” (P2, 23, Female). These accounts emphasize the important role of healthcare professionals in supporting informed decision-making and safer supplement use.

##### 5.9.4.3 Self-monitoring and perceived effectiveness

Participants commonly evaluated supplement effectiveness by monitoring changes in their mood, stress, sleep, energy, and physical symptoms. One participant explained, “I felt calmer after taking it, so I used it only when feel stressed” (P6, 38, Female), demonstrating intentional self-regulation based on perceived benefit. Similar self-monitoring was also reported for supplements other than ashwagandha. For example, one participant described continuing an iron supplement because symptoms associated with iron deficiency appeared to recur when its use was discontinued: “I noticed iron deficiency symptoms if I stop taking it, so I continue” (P2, 23, Female). These accounts illustrate individualized, trial-and-error approaches to supplement management, in which continued use was guided primarily by perceived bodily and psychological responses.

## 6 Discussion

This mixed-methods study examined Saudi adults’ knowledge, beliefs, and behaviors regarding ashwagandha. Most participants used ashwagandha for stress relief and emotional wellbeing, guided mainly by personal experience and social influence. However, many lacked adequate knowledge about dosage, long-term safety, and potential interactions. Informal sources such as social media, friends, and family outweighed formal education or healthcare guidance in shaping decisions.

### 6.1 Knowledge and information sources

Most participants were aware of ashwagandha’s perceived benefits but had limited knowledge about correct dosage, side effects, long-term safety, and possible drug interactions. Participants frequently obtained information about ashwagandha from social media, online reviews, family members, and friends, while healthcare professionals and scientific sources were consulted less often. This pattern suggests that informal and digital sources played an important role in increasing awareness and influencing participants’ perceptions of ashwagandha. However, the findings do not demonstrate that these sources were more influential than formal education or healthcare guidance at the individual level.

Previous ashwagandha use was associated with higher knowledge and more favorable attitudes, while frequency of use and a previous diagnosis of anxiety or another mental health condition were more closely related to attitudes than to knowledge. The qualitative findings helped contextualize these associations by showing that favorable personal experiences, perceived stress relief, and recommendations from social contacts often contributed to positive views of the supplement. Because the study was cross-sectional, these relationships should be interpreted as associations rather than evidence of causality.

These findings are consistent with earlier studies showing that digital platforms strongly influence perceptions of herbal and complementary medicine (Zaidi et al., 2022; Chandrasekhar et al., 2012). Social media platforms such as TikTok, Instagram, and YouTube are now major sources of health information, although the quality and accuracy of content remain inconsistent. Many participants trusted influencer recommendations and anecdotal experiences despite uncertainty regarding scientific evidence or safety.

Although many participants recognized ashwagandha’s role in stress reduction and relaxation, few understood its mechanisms, contraindications, appropriate dosing, or interactions. A systematic review by (Pratte et al., 2014) evaluated five human randomized controlled trials investigating ashwagandha as an intervention for anxiety or stress. The included studies generally reported greater improvements in anxiety or perceived stress outcomes among participants receiving ashwagandha than among those in the comparison groups. However, the authors emphasized that these findings should be interpreted cautiously because of heterogeneity in study designs and outcome measures and the unclear or high risk of bias across the included trials (Pratte et al., 2014). While research supports ashwagandha’s calming effects (Haslan et al., 2015), evidence-based understanding of safe use remains limited.

### 6.2 Attitudes, risk perception, and behavioral interpretation

Participants expressed mixed attitudes, balancing perceived benefits with concerns regarding risks. Qualitative findings indicated that participants perceived ashwagandha as potentially beneficial for stress reduction, emotional wellbeing, relaxation, and sleep. At the same time, they expressed concerns about emotional numbness, possible dependence, uncertainty regarding appropriate dosage, potential adverse effects, and the safety of long-term use.

These attitudes differ from findings in some Western populations, where adaptogens are often viewed more positively (Wiciński et al., 2025). Such differences may be related to variations in sociocultural context, health literacy, regulatory environments, and access to healthcare and evidence-based information. In the present study, social media and interpersonal influence also emerged as important factors shaping participants’ awareness and perceptions of ashwagandha.

Attitudes were strongly shaped by personal experience. Participants who reported improvements in stress, sleep, or mood were more likely to continue using ashwagandha, supporting previous evidence that personal experience frequently outweighs scientific knowledge in complementary medicine decisions (Khan et al., 2025; Benzie and Wachtel-Galor, 2011; Syed et al., 2022). Use patterns were largely intermittent and self-regulated, especially during periods of psychological distress, academic pressure, emotional exhaustion, or sleep disturbance, consistent with studies linking herbal supplement use to coping with stress and anxiety (Khan et al., 2025; Benzie and Wachtel-Galor, 2011; Syed et al., 2022).

The qualitative findings indicated that participants modified both the dosage and frequency of ashwagandha use according to perceived benefits, tolerance, adverse effects, and information obtained from online or informal sources. Participants reported taking ashwagandha primarily in capsule form, with daily doses of either 400 or 700 mg. The reported dosing practices varied among participants and appeared to reflect individualized, self-directed supplement use rather than use under consistent professional supervision.

Participants also reported access to different ashwagandha formulations in Saudi Arabia, including capsules, powders, teas, and extracts. Capsules were the most commonly used formulation of ashwagandha. However, three participants (15%) also mentioned tea or extract formulations and perceived them as potentially more potent or pure. These perceptions were subjective and were not supported by laboratory evaluation of product composition, concentration, purity, or bioavailability. The findings therefore highlight the importance of professional counseling regarding dosage, formulation, product labeling, and the risks associated with unsupervised use.

The present study relied on participants’ self-reported experiences and did not include clinical assessment, laboratory testing, or verification of reported benefits, adverse effects, dosages, or product composition. Therefore, participants’ accounts should not be interpreted as evidence of clinical effectiveness or safety. Nevertheless, these perceptions remain important because they may influence decisions regarding supplement initiation, continuation, discontinuation, dose adjustment, and healthcare-seeking behavior. The findings are discussed in relation to existing pharmacological and clinical literature solely to provide interpretive context rather than to clinically validate participants’ reports. Participants frequently reported uncertainty regarding side effects, dosing, and safety information. These findings reinforce the importance of routinely asking patients about herbal supplement use, particularly among individuals experiencing stress, anxiety, sleep disorders, or chronic illness (Chandrasekhar et al., 2012; Khan et al., 2025; Alamoudi et al., 2021; Bhatiasevi, 2024). Greater awareness regarding side-effect monitoring, patient–clinician communication, and evidence-based counseling on interactions and safe use is needed (Kim et al., 2023; Posadzki et al., 2013; Bonilla et al., 2021; Coope et al., 2026; Özduran et al., 2023; Alum et al., 2025). Healthcare providers should actively discuss supplement use with patients, especially as reliance on social media and multiple supplements continues to increase (Zaidi et al., 2022; Chandrasekhar et al., 2012; Posadzki et al., 2013; Bonilla et al., 2021).

### 6.3 Pharmacological context and clinical evidence

Ashwagandha contains several active compounds, particularly withanolides, which have demonstrated anxiolytic, anti-inflammatory, and stress-modulating properties. These compounds may influence the hypothalamic–pituitary–adrenal axis, alter cortisol responses, and modulate neurotransmitter systems involved in stress, anxiety, and relaxation, including gamma-aminobutyric acid signaling (Benzie and Wachtel-Galor, 2011). Concurrent use of other herbal supplements across different cultures has its own particular features determined by health beliefs, traditions, availability of herbal solutions, their expected effectiveness, and sources of health information. For example, in a study considering patients with cerebrovascular diseases, herbal products were employed mainly for alleviating symptoms. One of the factors influencing this choice was trust in herbal medicine, followed by the high price-to-value ratio of herbal solutions and their availability in the community. Participants’ accounts suggested that recommendations from healthcare professionals, together with the availability of herbal products and exposure to information through social and digital sources, may influence decisions regarding herbal-product use.” The local traditions were shown to influence people’s preferences regarding the choice of herbal remedies indicating the strong inclination of individuals towards the use of specific remedies based on their traditions (Coope et al., 2026).

The present findings regarding stress reduction and sleep improvement are biologically plausible and align with randomized controlled trials and systematic reviews demonstrating ashwagandha’s effectiveness in reducing anxiety, stress, and insomnia compared with placebo (Alsanie et al., 2026; Chandrasekhar et al., 2012; Pratte et al., 2014; Özduran et al., 2023). Nevertheless, concerns remain regarding product quality, dosage variability, and limited evidence on long-term safety, particularly in unsupervised community use.

Ashwagandha has long been used within Ayurvedic medicine for resilience, vitality, and emotional balance. Recently, however, its popularity has expanded globally through online marketing, wellness culture, and increasing interest in “natural” remedies. Within Saudi Arabia, rising interest in herbal and complementary medicine may reflect cultural traditions, holistic health preferences, and increasing stress associated with modern lifestyles, academic demands, and sleep problems.

Participants often viewed ashwagandha as part of a broader self-care strategy shaped by peer influence and online wellness trends. At the same time, concerns regarding scientific evidence, product quality, and regulation remained evident. This dual perception may explain participants’ ambivalent attitudes toward ashwagandha as both beneficial and potentially risky. These findings highlight the need for culturally sensitive health education, evidence-based counseling, and stronger pharmacovigilance systems in Saudi Arabia and similar settings (Zaidi et al., 2022; Başaran et al., 2022; Chandrasekhar et al., 2012; Kim et al., 2023; Posadzki et al., 2013; Bonilla et al., 2021; Coope et al., 2026; Özduran et al., 2023; Alum et al., 2025).

### 6.4 Predictors of knowledge and attitudes

The regression findings indicated that previous ashwagandha use was associated with greater knowledge and more favorable attitudes toward the supplement. Frequency of use was significantly associated with attitudes but not with knowledge, suggesting that repeated exposure may influence perceptions of ashwagandha more strongly than factual understanding. A previous diagnosis of anxiety or another mental health condition also showed a stronger association with attitudes than with knowledge; however, its association with knowledge did not reach statistical significance. In contrast, sociodemographic characteristics, chronic health conditions, and Concurrent use of other herbal supplements were not significant independent predictors of either outcome.

These findings suggest that personal experience with ashwagandha and psychological health needs may shape attitudes toward the supplement more strongly than demographic characteristics such as age, gender, education, or marital status. The weak but statistically significant positive correlation between knowledge and attitude scores further indicates that greater knowledge was associated with more favorable attitudes. Nevertheless, these relationships should be interpreted as associations rather than causal effects because of the cross-sectional study design.

The findings also highlight the need to improve public awareness of the safety profile of ashwagandha, including its potential interactions with prescription and over-the-counter medications. Previous studies have shown that individuals may use herbal products concurrently with conventional medications without consistently informing healthcare providers. This creates challenges for clinicians, who may be unable to recognize potential herb–drug interactions when patients’ herbal-product use is not routinely assessed. Research among patients attending pain clinics has similarly documented herbal-product use and emphasized the importance of routine assessment and patient education to promote medication safety (Rombolà et al., 2020).

### 6.5 Decision-making, safety concerns, and self-regulation

Participants commonly self-managed their supplement use through dose adjustments, temporary discontinuation, and side-effect monitoring. Few consulted healthcare professionals, relying instead on friends, online communities, and personal experimentation.

These findings align with World Health Organization recommendations promoting integration of traditional medicine into healthcare systems (Kim et al., 2023). Lack of professional guidance may increase the risks of misinformation, incorrect dosing, and unknown interactions. Ashwagandha was frequently combined with vitamins, minerals, herbal remedies, and fitness supplements, potentially increasing interaction risks and complicating symptom attribution (Posadzki et al., 2013; Bonilla et al., 2021). Various clinics might face difficulties concerning the interactions between herbs and medications, as patients often consume herbal products without notifying their healthcare providers. As noted by Özduran et al. (Fani Pakdel et al., 2021), a significant number of users possess limited understanding of side effects and possible interactions, and physicians may not always be informed about their patients’ use of Concurrent use of other herbal supplements The easy access to herbal products, inconsistent labeling, misinformation from media and commercial entities, and the growing use of multiple medications in patients with chronic illnesses all contribute to the heightened risk of interactions between herbs and drugs. To address these issues, clinicians need to regularly evaluate the use of herbal products and educate patients about safety and potential adverse effects.

The current findings should be interpreted in the context of participants’ reliance on social media and other informal digital information sources. The quantitative survey assessed broad categories of information sources rather than individual platforms; therefore, it was not possible to estimate separately the proportions of participants obtaining information from YouTube, TikTok, or AI-driven platforms. Nevertheless, the qualitative interviews provided examples of participants encountering ashwagandha-related information through specific social media platforms, including TikTok. These qualitative accounts show how online testimonials which is defined as personal statements, reviews, and anecdotal reports shared on social media, online forums, and other internet sources shaped participants’ expectations and decisions about using ashwagandha. For example, a post stating, “Ashwagandha helped me sleep better,” may influence others’ views of its potential benefits. Accordingly, the present findings support the influence of informal digital information generally, rather than any specific online platform.

Participants also demonstrated limited understanding of the regulatory oversight of ashwagandha products. As shown in Table 4 60% of participants answered the Saudi Food and Drug Authority (S FDA)-related knowledge item incorrectly, whereas only 40% provided the correct response. This finding indicates that a substantial proportion of participants may not clearly understand whether and how herbal supplements are regulated, evaluated, or monitored by the relevant national authority (Coope et al., 2026). Such misunderstanding may affect consumers’ assumptions about product approval, safety, effectiveness, and quality and may lead some individuals to interpret commercial availability as evidence that a product has undergone comprehensive clinical evaluation.

The current study did not directly assess the labeling accuracy, ingredient variability, contamination, batch consistency, or laboratory testing of ashwagandha products. Therefore, no conclusions can be drawn regarding the actual quality of the products used by participants. However, concerns about discrepancies between labeled and measured ingredients, variability across herbal preparations, and differences in product testing have been reported in the international literature (Özduran et al., 2023; Alum et al., 2025). These broader concerns are relevant to the present finding because limited regulatory knowledge may prevent consumers from recognizing that product quality and evidence of effectiveness can vary among manufacturers. The results therefore emphasize the need for clearer public communication regarding the meaning of regulatory registration, product approval, quality monitoring, and the limitations of safety and efficacy claims for herbal supplements. Pharmacists and other healthcare professionals may play an important role in helping consumers select reliable products and understand appropriate dosage, contraindications, and potential interactions.

### 6.6 Theoretical integration through the health belief model

The Health Belief Model served as an efficient tool in explaining how the experiences and beliefs of the participants influenced their decisions regarding ashwagandha (Posadzki et al., 2013; Bonilla et al., 2021). The participants used ashwagandha because of the interplay of many factors, not just their pharmacological knowledge. Participants’ decisions to use ashwagandha were influenced by their perceived need for symptom relief, expectations regarding its potential benefits, concerns about possible risks, and recommendations or information obtained from family members, friends, social media, and healthcare professionals. Their decisions were also shaped by their perceived self-efficacy, including confidence in selecting an appropriate product, determining how and when to use it, monitoring its effects, and deciding whether to continue or discontinue use.

Concerns about anxiety, stress, sleep disorder, and emotional problems were indicative of the perceived susceptibility and severity. People who paid attention to these issues were more likely to try ashwagandha.

Participants described several perceived barriers to the safe use of ashwagandha, including uncertainty about appropriate dosage, concerns about potential adverse effects and long-term safety, and limited consultation with healthcare professionals. Some participants also expressed uncertainty about combining ashwagandha with medications or other supplements. During the qualitative interviews, three participants mentioned tea or extract formulations and expressed subjective perceptions that these formulations might differ from capsules in potency or purity. However, the present study did not independently assess or compare the composition, concentration, purity, quality, or bioavailability of the available formulations. Although these barriers were not sufficient to deter subjects completely, they nevertheless caused cautious, sporadic consumption behavior, or self-adjustment of the intake. Cues to initiate or consider ashwagandha use were shaped primarily by social media content, recommendations from family members and friends, anecdotal accounts and user reviews, and participants’ previous personal experiences, whereas healthcare professionals were mentioned less frequently as sources of guidance. (Fani Pakdel et al., 2021).

Self-efficacy varied among participants. Some felt confident selecting among available ashwagandha formulations primarily capsule products determining their own dosage, and monitoring perceived benefits or adverse effects. Others expressed uncertainty and relied on social media, family members, friends, or other informal sources for guidance. Overall, the model suggests that participants’ decisions regarding ashwagandha use were shaped by how they balanced its perceived benefits against its perceived risks, together with the influence of personal experiences and informal social sources on their decision-making.

### 6.7 Qualitative findings and mixed-methods integration

Assessing traditional medicine like ashwagandha is a methodological challenge, given that herbal medicine often consists of a number of components and their amounts may differ according to cultivation, processing, extraction, formulation and storage conditions. Special relevance is to be attached to this aspect in a community setting since consumers may use medicines with different formulations and concentrations, and use them off-label as well.

Participants reported that their use of ashwagandha is largely individualized and intermittent, with users regulating their doses on their own. Participants’ decisions were based mainly on their personal experiences with commercially available ashwagandha formulations, particularly capsules, although some reported using powders, teas, or extracts. However, product packages, photographs, and detailed label information such as brand, ingredients, extract concentration, withanolide content, recommended dosage, and safety warnings were not systematically collected. Therefore, the study could not evaluate the accuracy or consistency of the labeling and composition of the products used.

A significant alignment was found between the quantitative data and qualitative data. The quantitative and qualitative data indicated that stress-related needs, personal experience, social influence, and informal information were the determinants for people’s ideas about ashwagandha and their willingness to use it. The qualitative findings indicated that participants commonly developed their understanding of ashwagandha through personal experiences and informal information sources rather than through scientific evidence or guidance from healthcare professionals. According to quantitative data, the previously used and the more often used ashwagandha was linked to positive attitudes toward the supplement. Interviews provided an explanatory solution. People who believe that ashwagandha can help them cope with stress, relax, sleep better, improve their mood, or general condition expressed more confidence in its effectiveness.

Both components also highlighted the importance of informal communication. The qualitative findings provided additional context for the quantitative results by showing how participants encountered and interpreted information about ashwagandha. Participants commonly reported learning about the product through social media platforms, particularly TikTok and YouTube, as well as through friends and family members. Information obtained from these sources appeared to shape their expectations regarding stress relief, relaxation, sleep, and the perceived safety of ashwagandha. These findings indicate that social and digital information sources may contribute to individuals’ awareness and decisions regarding complementary and herbal products and are consistent with previous research describing the growing role of digital health communication in complementary-medicine practices. These findings are consistent with previous research demonstrating the increasing influence of digital health communication on complementary-medicine practices (Chandrasekhar et al., 2012; Rombolà et al., 2020).

Some divergence was identified regarding the combined use of ashwagandha with other products. Survey responses indicated relatively limited concurrent supplement use, whereas interview accounts suggested that the use of ashwagandha alongside vitamins, other herbal products, or traditional remedies may have been more common than the quantitative results indicated. This discrepancy may reflect underreporting, social desirability bias, or differences in how participants understood and classified supplements and concurrent use.

Rather than merely confirming the quantitative results, the qualitative findings provided contextual insight into the statistical association between previous ashwagandha use and more favorable attitudes. Participants’ accounts suggested that perceived benefits and prior personal experiences may have contributed to positive perceptions of ashwagandha. The interviews also illustrated how informal information sources shaped decision-making and how differences in participants’ interpretation of “concurrent supplement use” may have limited its capture through structured survey questions. These qualitative findings should be interpreted as complementary explanations rather than evidence of causal relationships. This integration identifies specific areas for future research, including clearer measurement of digital information sources, explicit definitions of concurrent supplement use, and closer examination of consumers’ self-management and safety practices.

### 6.8 Clinical and public health implications

These findings have clinical and public health implications. Participants commonly used ashwagandha for stress, relaxation, emotional wellbeing, and sleep, often based on personal experience or informal information sources. This highlights the need for culturally appropriate, evidence-based education and greater involvement of healthcare professionals in promoting safe use. Therefore, healthcare professionals must take into account the use of herbal supplements when assessing patients showing symptoms of stress, anxiety, sleep disorder, etc.

Secondly, nurses, physicians, and pharmacists should proactively promote safe usage of ashwagandha and herbal supplements instead of simply waiting for side effects and complex interactions to happen. Healthcare professionals should offer preventive education before the herbal or dietary supplement is taken, ask their clients about herbal and dietary supplements they are taking, clarify and correct any misinformation, and explain the dosage, duration of the treatment, and any possible side effects and drug interactions before the client starts taking any herbal products (Wang et al., 2023; Amer and Mohammad, 2022). Community pharmacists and general medicine doctors are in a good position to provide consultations as they are rather easily accessible to the population.

Furthermore, healthcare facilities and authorities should also proactively integrate education about complementary medicine into daily patient counseling practice, healthcare training, awareness campaigns, and digital health initiatives (Wang et al., 2023; Amer and Mohammad, 2022). Such training must clearly define what to consume in terms of safety, regulation, dosage, symptoms that should be reported to healthcare professionals, etc.

Education has also to be culturally sensitive. In Saudi Arabia, it means recognizing that herbal remedies are widely accepted in society and that traditional beliefs should not be disregarded and personal experiences respected. Educational materials must deliver vital information in an unobtrusive, reliable manner using simple Arabic and English.

The findings highlight several methodological and regulatory challenges associated with research on Concurrent use of other herbal supplements Such products may contain multiple bioactive constituents, and variations in formulation, concentration, labeling, dosage, and manufacturing standards may affect their safety and reported effectiveness. These ingredients can differ depending on the growing conditions, extraction technologies, processing methods, and storage conditions. This means that these products are harder to study with the help of conventional research methods (Benzie and Wachtel-Galor, 2011; Amer and Mohammad, 2022; Marginean, 2025). The participants of the study used herbal remedies in a way that corresponded to their individual needs and capabilities and was not regulated by any standards.

The mixed-methods design enabled the identification of both patterns of use and the personal experiences that influenced participants’ decision-making regarding ashwagandha. Overall, the results of the study suggest that it is important to provide comprehensive counseling to the public, use culturally appropriate educational programs, monitor the issues of safety in herbal medicine, and communicate evidence-based information concerning herbal supplementation (Marginean, 2025).

## 7 Strengths of the study

This study is the first to examine how Saudi Arabian adults perceive, understand, and use ashwagandha in Arab culture. A major strength of this study was the use of a convergent mixed-methods design, which enabled the integration of quantitative patterns with participants’ personal experiences and perceptions. The relatively large and diverse sample also enhanced the breadth of the findings, while the qualitative component provided contextual insight into the social, cultural, and informational factors associated with ashwagandha knowledge, safety perceptions, and use.

## 8 Limitations

There are some limitations to this study that should be kept in mind. The researchers used convenience and purposive sampling, which may cause selection bias. The sample might not fully represent the wider population. In terms of demographics, young females formed the bulk of the sample, as 79.7% of participants were under 30 years of age and 78.9% were females. This result probably reflects the online recruitment techniques that could have favored women and young adults in learning and filling out the survey. As a result, the outcomes might not be a true representation of the views of older population groups and males in Saudi Arabia. People interested in herbal supplements, health, or social media health topics may have been more likely to take part. Recruiting participants online may have excluded those with limited internet access or lower digital skills. It may also exclude those who are less active on online platforms.

Due to the limitations, the findings cannot be generalized to the entire Saudi population and should be interpreted with caution, they apply only to the participants in this study. The study identified links between knowledge, attitudes, safety perceptions, and ashwagandha use. However, as a cross-sectional study, it cannot determine causal relationships. Even with these limits, combining quantitative and qualitative methods provided valuable early insights: Saudi adults’ use of ashwagandha is shaped by perceived health benefits, cultural beliefs, social influence, and personal experiences. Most participants linked ashwagandha to stress reduction, relaxation, better sleep, and emotional wellbeing, while their decisions relied more on personal experiences and informal sources than on scientific evidence.

## 9 Conclusion

The findings revealed substantial gaps in participants’ knowledge, particularly regarding appropriate dosage, potential adverse effects, drug–supplement interactions, and the long-term safety of ashwagandha. Although most participants expressed positive attitudes, many relied on social media, anecdotal accounts, or advice from non-expert sources. Participants recognized the potential benefits of ashwagandha while also expressing concerns about its possible risks and long-term safety. These findings emphasize the need for clear public health education and accessible, evidence-based information to support its safe and informed use. Greater involvement of healthcare professionals, strengthened safety monitoring, and open communication between patients and healthcare providers are also essential.

## 10 Recommendations

### 10.1 Research recommendations

- The study points to the need for larger, long-term research to better understand ashwagandha’s safety, effectiveness, use, and interactions across different groups, as well as for additional studies tracking changes in knowledge, attitudes, and use over time.
- Explore healthcare providers’ perceptions of herbal products to inform better education, improve safety, and encourage effective patient-provider communication.
- Assess the validity, quality, and safety of ashwagandha products, including their interactions with prescription drugs, to ensure consumer safety.
- Evaluate effective educational interventions that enhance knowledge about herbal supplements, including ashwagandha, and address key knowledge gaps.

### 10.2 Practical recommendations

#### 10.2.1 Healthcare provider consultation

Provide consumers with clear information about ashwagandha, including its features, proven health benefits, potential risks, and proper dosing. This education should come from primary healthcare providers such as pharmacists and family doctors.

#### 10.2.2 Awareness of regulatory and safety issues

- Educate the public on how ashwagandha differs from regulated pharmaceuticals. Ashwagandha is classed as a dietary supplement. The Saudi Food and Drug Administration regulates the quality, labeling, and marketing of products in Saudi Arabia. Also, ashwagandha brands can vary widely in their chemical composition and effects.
- Tell the public how to use ashwagandha safely. This means not exceeding the recommended dose and following label instructions. People should know when ashwagandha should not be used, for example, during pregnancy or with certain illnesses.
- Make people aware of the laws that govern the use of ashwagandha. Advise choosing brands approved by the SFDA. Encourage reporting of any side effects experienced.

#### 10.2.3 Educational materials/public health campaigns

- Create and share trustworthy materials, such as brochures, social media posts, workshops, and presentations, that explain the benefits and risks of ashwagandha and how to use it properly.
- Run public health campaigns to correct online misinformation and encourage people to track their own ashwagandha use.

#### 10.2.4 Monitoring and self-regulation

- Encourage users to pay attention to how ashwagandha affects them, including both benefits and any negative changes.
- Individuals who experience adverse effects while using ashwagandha together with other herbal or nutritional supplements should discontinue self-directed dose adjustments and consult a qualified healthcare professional for assessment and guidance on safe supplement use.

## Data Availability

The raw data supporting the conclusions of this article will be made available by the authors, without undue reservation.
